# Probing for Intentions: Why Clocks Do Not Provide the Only Measurement of Time

**DOI:** 10.3389/fnhum.2019.00068

**Published:** 2019-03-12

**Authors:** Ceci Verbaarschot, Pim Haselager, Jason Farquhar

**Affiliations:** Centre for Cognition, Donders Institute for Brain, Cognition and Behaviour, Radboud University, Nijmegen, Netherlands

**Keywords:** action, awareness, EEG, ERD, intention, movement, probe, RP

## Abstract

Having an intention to act is commonly operationalized as the moment at which awareness of an urge or decision to act arises. Measuring this moment has been challenging due to the dependence on first-person reports of subjective experience rather than objective behavioral or neural measurements. Commonly, this challenge is met using (variants of) Libet's clock method. In 2008, Matsuhashi and Hallett published a novel probing strategy as an alternative to the clock method. We believe their probe method could provide a valuable addition to the clock method because: it measures the timing of an intention in real-time, it can be combined with additional (tactile, visual or auditory) stimuli to create a more ecologically valid experimental context, and it allows the measurement of the point of no return. Yet to this date, the probe method has not been applied widely - possibly due to concerns about the effects that the probes might have on the intention and/or action preparation processes. To address these concerns, a 2 × 2 within-subject design is tested. In this design, two variables are manipulated: (1) the requirement of an introspection report and (2) the presence of an auditory probe. Three observables are measured that provide information about the timing of an intention to act: (1) awareness reports of the subjective experience of having an intention, (2) neural preparatory activity for action, and (3) behavioral data of the performed actions. The presence of probes was found to speed up mean action times by roughly 300 ms, but did not alter the neural preparation for action. The requirement of an introspection report did influence brain signals: reducing the amplitude of the readiness potential and increasing the desynchronization in the alpha and beta bands over the motor cortex prior to action onset. By discussing the strengths and weaknesses of the probe method compared to the clock method, we hope to demonstrate its added value and promote its use in future research.

## 1. Introduction

Having an intention to act is commonly operationalized as the moment at which awareness of an urge or decision to act arises (Libet et al., 1983; Lau et al., 2007; Soon et al., 2008; Fried et al., 2011; Tabu et al., 2015; Alexander et al., 2016). Measuring this moment has been challenging due to the dependence on first-person reports of subjective experience rather than objective behavioral or neural measurements (Wolpe and Rowe, 2014; Haggard, 2019). A popular method to measure the timing of an intention to act is the clock method of Libet et al. (1983). This method instructs participants to look at a clock and remember its configuration as soon as they experience an intention to act. This configuration is to be remembered and reported after the action has been performed. Variants of this paradigm use a stream of letters (Soon et al., 2008, 2013; Bode et al., 2011) instead of a traditional clock. Although this method is widely applied (Dominik et al., 2018; Saigle et al., 2018), it has been criticized repeatedly (Haggard, 2008; Nachev and Hacker, 2014; Navon, 2014; Wolpe and Rowe, 2014). Major critiques concern the requirement of constant introspection, the *post-hoc* nature of the intention reports and the ecological validity of the experimental task.

In 2008, Matsuhashi and Hallett came up with an alternative to the clock method. They invented a novel probing strategy to measure the experienced timing of an intention to act. Their strategy uses auditory probes that are presented to a participant at random points in time. These probes trigger a report on the awareness of an intention to act through a behavioral response. When a probe is presented and the participant is experiencing an intention, they need to refrain from acting (i.e., *veto*) and wait. Alternatively, when a probe is presented when they are not intending to act, they should simply *ignore* the probe and continue their self-paced actions. By comparing the timing of probes and consequent actions, one can determine during which time period the participant was aware of their intention to act.

We believe the probe method of Matsuhashi and Hallett (2008) provides a valuable addition to the popular clock method of Libet et al. (1983) for several reasons. First of all, the probe method does not require constant introspection: participants need to perform introspection only for a brief moment in response to a probe. Secondly, the probes are presented during action preparation, measuring the timing of an intention to act in real-time rather than *post-hoc* after action performance. Thirdly, the probe method can easily be used in combination with other visual or tactile stimuli. This provides the opportunity to use this method within a more complex environment and study a more ecologically valid experimental task. This way, the probe method can broaden our methodological repertoire so we can study intentional actions under various circumstances. Fourth, in addition to the timing of an intention to act, the probe method measures the *point of no return* (Matsuhashi and Hallett, 2008). This point of no return indicates until what time one is still able to veto an intended act.

Although the probe method of Matsuhashi and Hallett provides a valuable addition to the conventional clock method, it has not been applied widely. To the best of our knowledge, only one investigation (by us) has used this method since (Verbaarschot et al., 2016). The vast majority of researchers use the clock method to investigate the timing of an intention: Alexander et al. (2016), Banks and Isham (2009), Bode et al. (2011), Fried et al. (2011), Douglas et al. (2015), Haggard and Eimer (1999), Haggard et al. (2002), Keller and Heckhausen (1990), Miller et al. (2011), Jo et al. (2014), Lau et al. (2007), Rigoni et al. (2011), Schlegel et al. (2013), Schneider et al. (2013), Sirigu et al. (2004), Soon et al. (2008), Soon et al. (2013), Tabu et al. (2015), Wohlschläger et al. (2003), etc. Perhaps this is due to the fact that, while the clock method is not without problems, the probe method has some concerns of its own. These concerns mainly involve the effect that the probes might have on the experienced awareness of an intention to act, the timing of the performed actions and the underlying neural activity (as described in detail below). In order to address these concerns, a 2x2 within-subject design is tested. In this design, two variables are manipulated: (1) the requirement of an introspection report and (2) the presence of an auditory probe.

Three observables are measured that provide information about the timing of an intention to act: (1) awareness reports of the subjective experience of having an intention, (2) neural preparatory activity for action, and (3) behavioral data of the performed actions. The measured intention reports can consist of a *post-hoc* report on the vividness of an experienced intention, analog to the required constant introspection of the clock method (Libet et al., 1983). Alternatively, it can consist of the response to an auditory probe (i.e., ignore the probe or veto the action), as used in the probe method of Matsuhashi and Hallett (2008). The neural preparatory activity for action is recorded using an electro-encephalogram (EEG). Both the readiness potential (RP) and event-related desynchronization in the alpha (8–12 Hz) and beta (13–30 Hz) bands over the motor cortex prior to action are investigated. Both signatures have been reported to correlate with voluntary movement in previous research (Kornhuber and Deecke, 1965; Pfurtscheller and Aranibar, 1979; Libet et al., 1983; Doyle et al., 2005; Shibasaki and Hallett, 2006; Bai et al., 2011; Lew et al., 2012; Khalighinejad et al., 2018). The performed actions are measured through the timing of button presses and an electro-myogram (EMG) of the relevant arm-muscles.

*Post-hoc*, we investigated the brain activity prior to ignored and vetoed probes. When a probe is ignored, it means that the participant did not experience an intention to act at probe onset. We know that the RP has its onset up to 2s prior to action, whereas the awareness of an intention is reported up to 1.5s prior to action using the probe method (Matsuhashi and Hallett, 2008). This means that if a participant ignores a probe, one would expect no or a very weak RP prior to probe onset. However, when a probe is vetoed and the participant was experiencing an intention to act, one would expect to see an RP prior to probe onset. In this case the RP is time-locked to probe onset, therefore we expect it to be less pronounced (i.e., smaller amplitude) than when it would be time-locked to action onset. If we find an RP prior to vetoed probes and not prior to ignored probes, this would provide further credence to the probe method as an accurate tool for measuring the timing of an intention to act.

Before going into the details of our experiment, the next section (2) will provide additional background on the clock and probe methods. Section 3 describes our experiment which quantifies the concerns about the probe method by assessing the individual effects of the manipulated variables on each of the observables. Section 4 will compare the strengths and weaknesses of the probe method to those of the clock method. By addressing concerns and explicating the added value of the probe method, we hope to promote its use in future research.

## 2. Comparing Probes and Clocks

**Figure 1:**
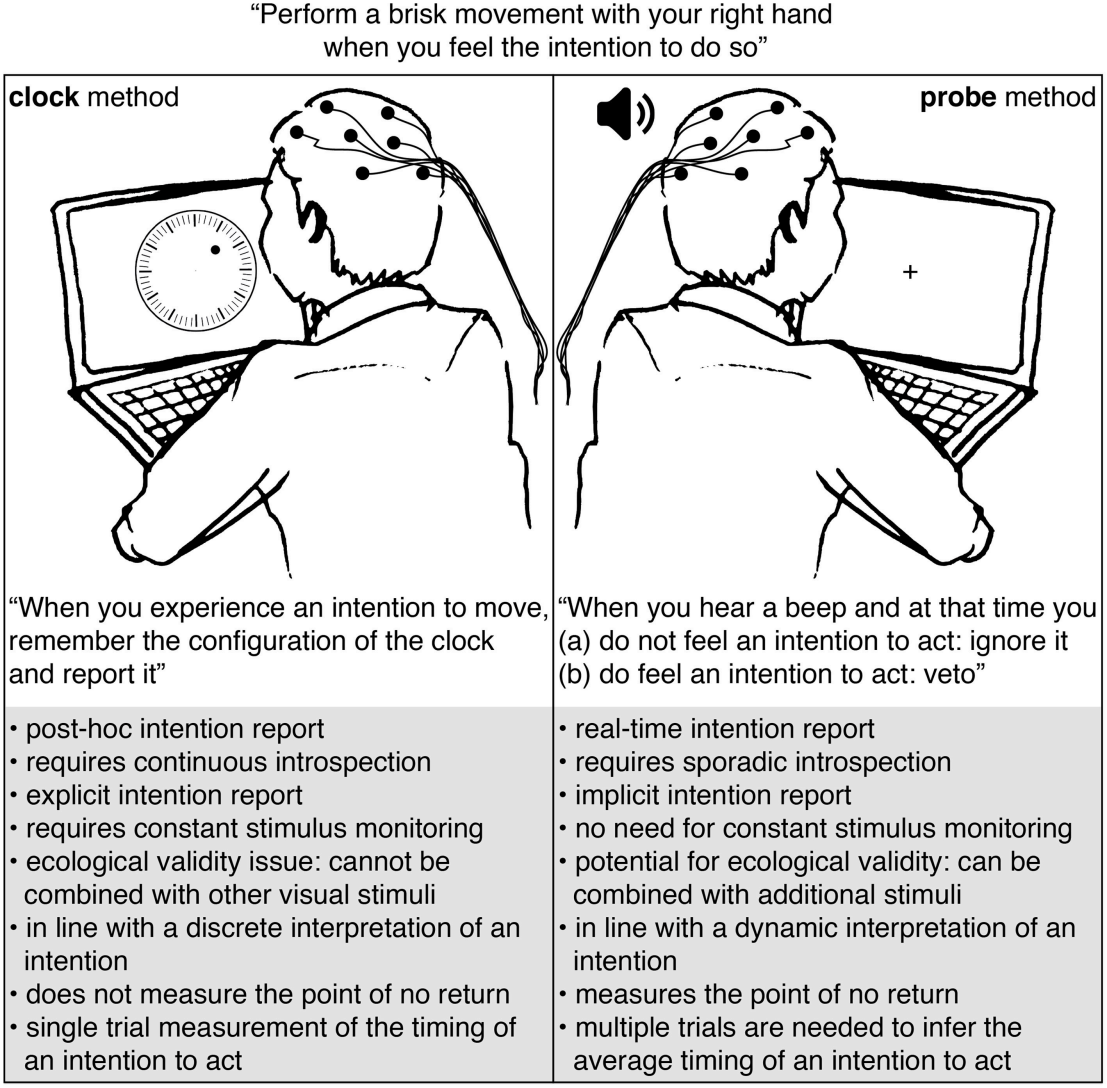
Comparison of the clock and probe methods.

Participants in studies that use the clock or probe method usually perform a similar motor task: a spontaneous action (e.g., a button press or brisk flexion of the hand) that is made by the participant whenever they experience an intention to do so. The difference between these methods is in the way they collect a report on the timing of an intention to act: see Figure 1. The clock method instructs participants to remember and report the configuration of a clock at the time of their experienced intention. The probe method uses auditory beeps to probe the participant at different moments in time for their awareness of an intention.

The probe and clock methods each have their strengths and weaknesses. First of all, the clock method requires participants to remember and report the onset of their intention after action performance Libet et al. (1983). This *post-hoc* method of gathering first-person reports seems prone to inaccuracies (Wolpe and Rowe, 2014). Moreover, the reported intention timings seem to be heavily influenced by the perceived action onset and/or the consequences of acting (Banks and Isham, 2009). The probe method measures the awareness of an intention on the spot. Participants need to respond immediately to a probe and have no need to retain the exact onset of their intention to act. Furthermore, since the awareness of an intention is measured prior to action performance, its timing cannot be influenced by the act itself or any of its potential consequences.

Second, the clock method requires continuous introspection: participants need to tune into their conscious experience to detect the slightest trace of an urge to act. This requirement seems to have an effect on the neural signatures that can be observed at that time (Lau et al., 2007). The probe method requires sporadic introspection: participants need to perform introspection for a brief moment in time in response to a probe. This happens once during a trial at most.

Third, the clock method requires an explicit intention report from a participant: participants are instructed to track the onset of their intention to act and remember and report its timing. Explicit awareness of an intention to perform a spontaneous (motor) action is not something we usually exhibit in our daily life. Requiring this awareness seems quite artificial. In contrast, the probe method uses the behavioral response to a probe to infer the time course of an intention to act. This implicit intention report softens the constraints on the level of awareness that is needed to perform the task. This situation seems similar to one in everyday life where we can explain our intentions when asked by someone.

Fourth, the required type of intended action that is studied by the clock and probe methods does not seem ecologically valid. Most investigations involve simple spontaneous actions like a self-paced hand movement (Libet et al., 1983; Sirigu et al., 2004; Matsuhashi and Hallett, 2008; Schlegel et al., 2013; Douglas et al., 2015) or a decision to add or subtract a number (Soon et al., 2013; Wisniewski et al., 2016). An alternative (more ecologically valid) task is difficult to find for the clock method. This is mostly due to the fact that it is difficult to combine the clock method with additional stimuli because focus and concentration is needed to observe a visually presented clock and remember the time of the experienced intention to act. In contrast, the probe method can easily be used in combination with other visual or tactile stimuli. This provides the opportunity to investigate the timing of an intention to act while performing actions that could be performed in everyday life. For instance, a recent study by Khalighinejad et al. (2018) made adaptations to a conventional moving-dot task in order to measure meaningful spontaneous hand movements. The target actions consist of voluntary decisions to skip a trial. Their design could be used in combination with the probe method to gain information on the timing of the intention to skip.

Fifth, the clock method seems in line with a discrete interpretation of an intention to act, whereas the probe method seems more in line with a dynamic one. The clock method asks participants to remember and report the moment at which they are aware of an urge or decision to act. This seems to assume that an intention is a discrete mental state that “pops up” in a participants mind at a specific moment in time, or at least that the awareness of that intention occurs at a discrete time (Uithol et al., 2014). The probe method questions the participant across a range in time, allowing a variety of moments at which one is aware of an urge or decision to act. Furthermore, the average onset of an intention to act measured with the probe method seems to be much earlier (about 1.5 s prior to action performance; see Matsuhashi and Hallett, 2008; Verbaarschot et al., 2016) than when it is measured with the clock method (about 0.15 s prior to action; see Libet et al., 1983; Haggard and Eimer, 1999; Haggard et al., 2002; Sirigu et al., 2004; Banks and Isham, 2009; Bode et al., 2011; Fried et al., 2011; Soon et al., 2013; Jo et al., 2014; Douglas et al., 2015; Tabu et al., 2015; Alexander et al., 2016). As argued previously (Verbaarschot et al., 2016), these findings fit better with the interpretation of an intention as a dynamic process rather than a discrete mental state. Unlike the clock method, the probe method can measure different stages in this process. However, the success of this method does come at a cost: quite a large amount of trials (±300) are required to get a good estimate of the awareness of an intention to act for a single participant. In contrast, the clock method provides a single-trial estimate of the onset of an intention.

Sixth and last, in addition to the timing of an intention to act, the probe method measures the point of no return (Matsuhashi and Hallett, 2008). When a probe is presented close to action onset, participants can no longer veto their action. According to Matsuhashi and Hallett, this inability to refrain from acting in response to a probe occurs around 0.13 s prior to action onset. The point in time at which this happens is referred to as the point of no return. Comparing the point of no return to the average brain activity at that point in time provides the opportunity to examine the stages of neural preparation for action after which action execution becomes irreversible. Schultze-Kraft et al. (2016), who found the point of no return at 200 ms prior to action onset, show that even after the onset of the Readiness Potential and alpha/beta ERD one can still veto an intended act. The clock method does not allow for any such analyses as it is unable to capture the point of no return.

**Figure 2:**
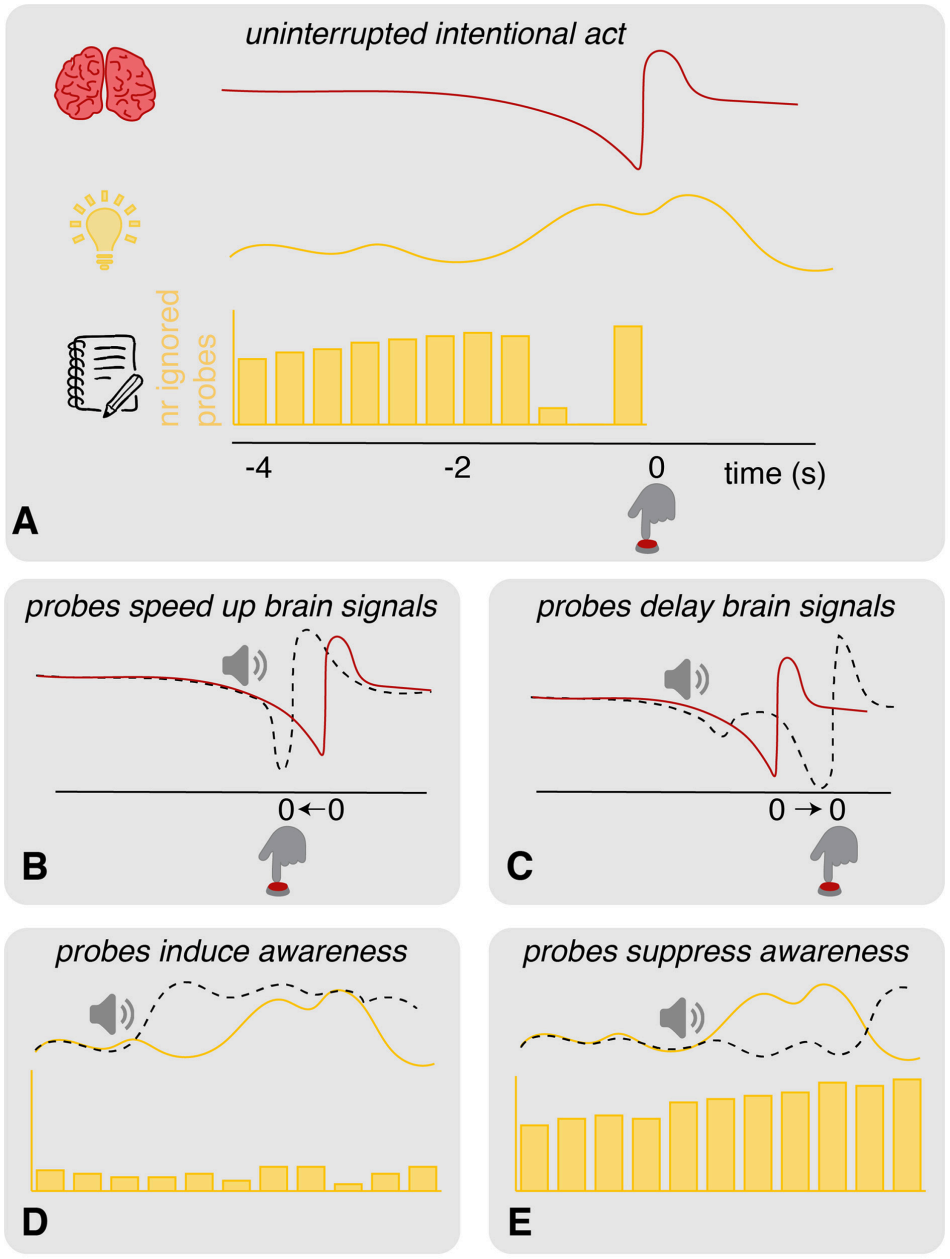
Illustration of hypotheses. **(A)** Schematic overview of the (top: brain) brain processes (e.g., RP), (middle: light bulb) experienced intentions and (bottom: notebook) intention reports (e.g., through veto and ignore responses to probes) prior to a spontaneous act. The gap in the distribution of ignored probes is due to vetos: prior to action onset, participants are aware of their intention and perform a veto in response to any probes that are presented at this time. Shortly prior to action onset, ignored probes reappear in the distribution: at this point, probes are presented so close to action onset that participants are unable to veto. **(B)** Probes could speed up brain signals by pushing the neural activity over the threshold for action. This should speed up actions as well. **(C)** Probes could delay brain signals, bringing the neural activity back to baseline. This should delay the actions. **(D)** Probes could induce awareness of intending to act. In this case, probes would often elicit a veto response, causing a sparse distribution of ignored probes that lacks a clear gap. **(E)** Probes could also suppress the awareness of intending to act. In this case, the distribution of ignored probes closely resembles the underlying distribution of scheduled probes. A clear gap due to consistent veto responses is missing.

Although the probe method seems to be a valuable addition to the clock method, it also raises some concerns. The requirement of an introspection report and the presence of a probe could potentially disrupt the “natural” process of intending to act in unknown ways (see Figure 2A). We have identified six possible scenarios that would invalidate the probe method as a tool to measure the timing of an intention to act ^1^:

1. *Probes speed up brain signals*: in an experimental context in which spontaneous actions are performed in absence of a clear external stimulus on when to act, intentions to act may be based largely on spontaneous fluctuations in neural activity (Schurger et al., 2012). Whenever the neural activity crosses a certain threshold, this results in an action. Threshold crossing tends to happen at crests in the ongoing neural fluctuations. Probes may affect these fluctuations and push them over the threshold for action performance (see Figure 2B). This influence may happen irrespective of the current stage of development of the RP and alpha/beta ERD. If this is the case, we expect to find more variance in these neural signatures in conditions with probes (*sound* + *probe*) compared to those without (*control* + *introspect*). On the other hand, the neural signatures may be susceptible to probes only during a specific stage in their development. If this is the case, we expect to find a later onset of these signatures relative to action performance in conditions with probes compared to those without. Irrespective of these two cases: if probes speed up brain signals, actions should speed up as well. Therefore, we expect that in both these cases actions will be faster in conditions with probes than those without. It could be the case that probes affect brain signals only if participants should pay attention to them. If this is the case, we expect to find the differences described above in the *probe* condition only.
2. *Probes delay brain signals*: rather than pushing the neural fluctuations over the threshold for action performance, probes may bring neural activations back to baseline level (see Figure 2C). If this is the case, we expect to find more variance in the RP and alpha/beta ERD in conditions with probes (*sound* + *probe*) compared to those without (*control* + *introspect*). Moreover, we expect to find slower actions in conditions with probes compared to those without. Again, these effects may be specific to conditions in which the probe matters for the task at hand. In that case, we expect to find these differences in the *probe* condition only.
3. *Probes induce awareness*: the presentation of a probe may enhance the awareness of an intention to act or even cause an intention to act (see Figure 2D). If this is the case, almost all probes in the *probe* condition should result in a veto response. This would cause the distribution of ignored probes to look sparse.
4. *Probes suppress awareness*: probes may also suppress awareness of an intention to act (see Figure 2E). In this case, probes should almost never result in a veto response in the *probe* condition. The distribution of ignored probes should look very similar to the distribution of scheduled probes.
5. *Veto influences action*: participants may dislike the required veto response and may therefore attempt to act before a probe is presented. In this case, actions should be performed faster in the *probe* condition compared to the other conditions.
6. *Inaccurate intention report*: participants may simply not be able to report their intentions to act using the probes. In this case, veto's are expected to be performed randomly in response to a probe. In this case, the distribution of ignored probes of the *probe* condition should look quite similar to the distribution of scheduled probes: there is no clear time range during which vetos are consistently performed.

To quantify these six concerns, each scenario is tested using the methods described in the next section.

## 3. Materials and Methods

### 3.1. Participants

The experiment was conducted in accordance with the ethical standards provided by the 1964 Declaration of Helsinki. The study protocol was approved by the local Ethics Committee of Faculty of Social Sciences of the Radboud University Nijmegen. A total of 21 healthy participants (15 females, mean age: 26 years old, youngest participant: 19 year old, oldest participant: 55 years old) volunteered to perform the experiment with their written informed consent. All participants were right-handed and had normal or corrected-to-normal vision. Participants received €25 or 2.5 course credits for their participation.

Five participants were excluded from the analysis. One participant reported to suffer from a brain disease that affects the amount of blood vessels present in the brain. Since it is unknown how this disease might affect their brain activity or behavior in this experiment, it was decided to exclude this participant from further analysis. Another participant reported to be nauseous during the experiment and could not sit still. Due to the large amount of resulting movement artifacts in the EEG data, this participant was excluded from further analysis. Similarly, many movement artifacts were found in the data of one other participant, leading to their exclusion from further analysis. Two participants did not follow instructions correctly, as was apparent from their answers to a post-experiment questionnaire. They were also excluded from analysis. The data of the remaining 16 participants was analyzed.

### 3.2. Experimental Procedure

The experiment consisted of a 2 × 2 within subject-design in which the following variables were manipulated: (1) the requirement of an introspection report and (2) the presence of an auditory probe. The main task of the participants, underlying each of the four conditions, was to press a button with the index finger of their right hand whenever they wanted to, similar to Libet et al. (1983) and Matsuhashi and Hallett (2008). Participants were instructed not to plan their actions, but press the button as soon as they felt an intention to do so. While performing these self-paced spontaneous actions, participants were instructed to look at a fixation cross that was displayed at the center of a computer screen. As long as the fixation cross was present, participants were instructed to relax, rest their arms and hands in between button presses and blink as little as possible.

When there are no reasons for deciding when to act other than a spontaneous intention to do so, it is challenging to prevent participants from acting as soon as possible (i.e., within the first 2 s of a trial). In Libet-type experiments this is done by instructing participants to wait for at least one full revolution of the clock before acting (Libet et al., 1983; Haggard and Eimer, 1999). Matsuhashi and Hallett (2008) instructed participants to perform their actions at intervals of roughly 5–10 s, without planning their actions or keeping time. When the action intervals were too long or short, participants were notified by the experimenter. Because details are missing on exactly when and how the participants were notified during the experiment of Matsuhashi and Hallett (2008), we achieved the action interval of 5-10 s using trial-by-trial color feedback: immediately at every button press, the color of the fixation cross changed for 1 s before the cross disappeared. If it turned blue, the action was made too slow; if it turned red, the action was made too fast; and if it turned green the action timing was perfect. The participant was instructed to adjust the timing of their button-presses depending on the color feedback. In this way, participants had no need to keep track of time, but could rely on the color feedback. The trained action timing provided a window of opportunity of about 5 s. During this time window, participants were free to perform a spontaneous act. This window ensured that there would be enough data for the subsequent EEG analysis and sufficient time to present a probe prior to action onset.

**Figure 3:**
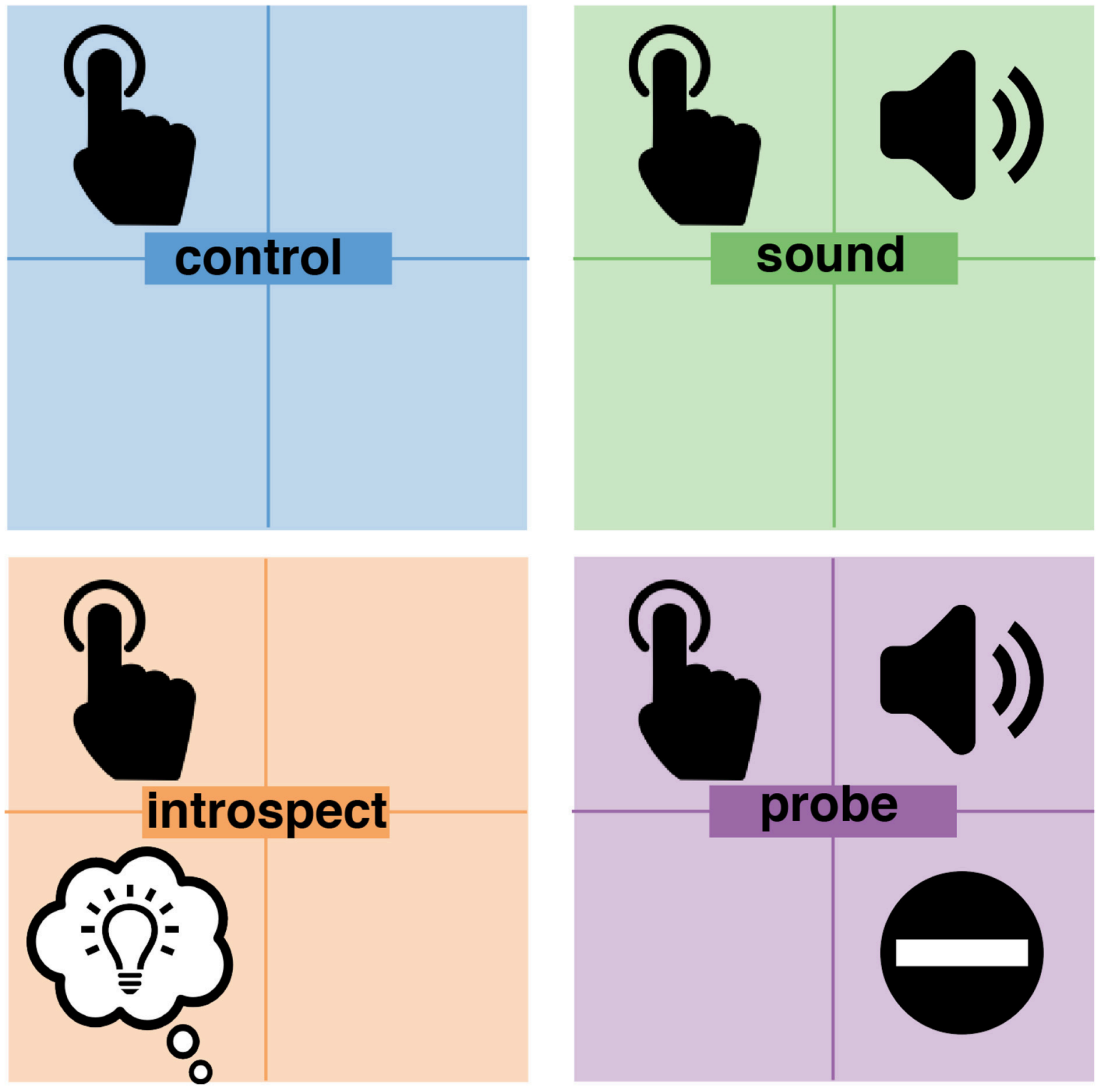
Overview of experimental conditions. In all conditions, participants were pressing a button at their own pace. In addition, the *sound* and *probe* conditions presented an auditory probe to participants at pseudo-random moments in time. The *introspect* and *probe* conditions both required a report on the awareness of an intention to act. In the *introspect* condition, participants needed to report *post-hoc* how vividly they experienced their intention to act. In the *probe* condition, participant needed to veto their action in response to a probe when at that time they were aware of their intention to act.

On top of the main task of performing self-paced button presses, each of the two independent variables were individually manipulated. This resulted in the following four experimental conditions (visualized in Figure 3):

1. *Control*: participants performed the main task of pressing a button at their own pace roughly every 5–10 s. An introspection report on their experienced intention was not required. This was the most basic condition as it consisted solely of the performance of spontaneous voluntary actions. With no additional stimuli or mental tasks, this condition provided pure control data for the timing of intended actions and their preceding neurological signals.
2. *Sound*: in addition to the main task, an auditory probe was presented at random times. Participants were instructed to ignore this probe completely because it has no importance to the experiment. Again, an introspection report on their experienced intention was not required. This condition allowed the investigation of any potential effects of the added auditory stimuli on the neural preparation for action, the awareness of an intention and the action itself.
3. *Introspect*: This condition did not involve any probes, but did require an introspection report. In addition to the main task, participants were instructed to focus their attention on the first moment at which they felt the urge to press the button. Immediately at every button press, the following multiple-choice question was presented: “How did you experience your intention to act?”. Participants could answer this question by pressing one of three buttons corresponding to the following answer options: “vivid and conscious,” “a vague feeling of wanting” or “pressed the button without thinking about it.” To prevent action preparation prior to the presentation of this question, the order of these answer options was set randomly at the start of each trial. Using these instructions and questions, participants were required to maintain a constant meta-awareness of their intention to act. This requirement mimicked the level of introspection required in a Libet-type experiment (Libet et al., 1983) and allowed the investigation of any effects of the pure introspection task - without the additional visual stimuli and memory tasks required by the clock method. Color feedback on action timing was provided immediately after answering the multiple-choice question.
4. *Probe*: in addition to the main task, an auditory probe was presented at random times and an introspection report was required. When the probe is presented while (1) they had an intention to act: they should *veto* the intended act (i.e., not press the button) and wait for the fixation cross to disappear. Alternatively, when a probe is presented while (2) they did not have an intention to act: they should *ignore* the probe and press the button whenever they wanted to. These instructions were a direct replication from Matsuhashi and Hallett (2008). Whenever a trial ended without a button press, the question “did you intend to act at the time you heard the beep?” was presented. The participant could answer this multiple-choice question with either “yes” or “no.” To prevent action preparation prior to the presentation of this question, the order of these answer options was determined randomly at the start of each trial. This question was presented in order to distinguish a veto from the absence of a button press (i.e., the trial ended before the participant experienced an intention to act). The subjective experience of an intention was reported indirectly through the behavioral response (i.e., veto or ignore) to a probe and was confirmed by answering the question at the end of a trial. This was the core condition that implemented the full probe method. Color feedback on action timing was provided immediately at each button press or, in case a trial ended without a button press, after answering the multiple-choice question.

At the end of the experiment, participants 8 to 21 completed a short questionnaire in order to gain more insight into their subjective experience during the experiment. The questionnaire consisted of the following seven questions (translated from Dutch):

1. Did you act spontaneously?
  - Yes, I pressed the button as soon as I wanted to
  - No, long before I pressed the button, I already decided to act
2. Was the difference between the tasks clear?
  - Yes
  - No
3. What did you think about the beeps?
  - Annoying
  - Neutral
  - Stressful
  - Other: …
4. Was it difficult to determine whether you had an intention to act at the time you heard the beep?
  - Yes
  - No
  - Sometimes
5. How did you decide whether you could press the button after you heard a beep?
6. Was there a clear difference between the moment at which you had an intention to press the button and the moment at which you pressed the button?
  - Yes, the intention to press the button was clearly distinguishable from the button press itself
  - No, the intention to press the button occurred at the same time as the button press
  - Other: …
7. Did the beeps influence your intention to press the button?
  - No
  - Yes, because: …

### 3.3. Stimuli

The participant was seated on a comfortable chair in front of a table inside a quiet room. The instructions and visual stimuli were displayed using a 17 inch TFT screen with a resolution of 800 by 600 pixels and a refresh rate of 60Hz that was placed roughly at 70cm directly in front of the participant. In-ear headphones were used to present the auditory probes. A button box containing a total of four buttons was used to perform the self-paced button presses and answer the questions in the *introspect* and *probe* conditions. The experiment was run in BrainStream ^2^.

**Figure 4:**
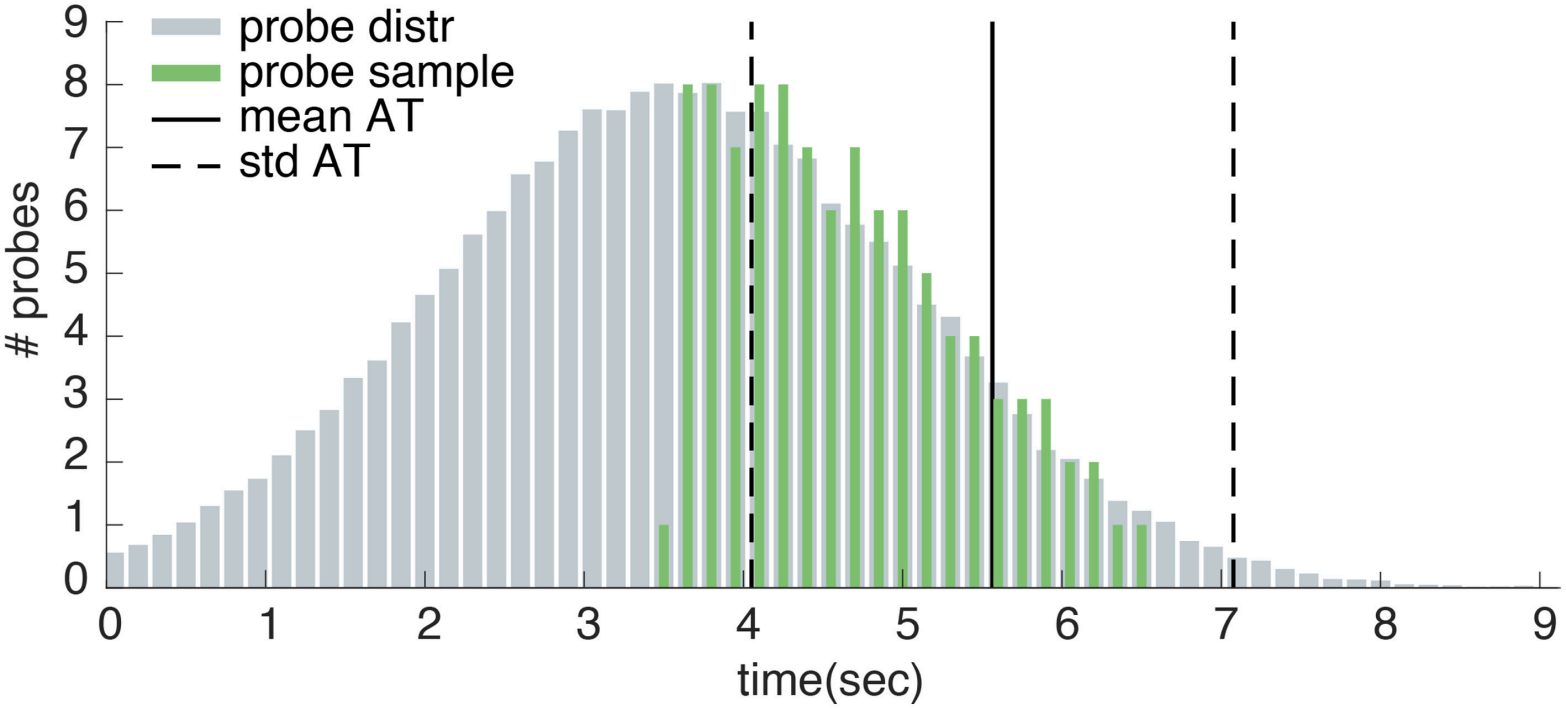
Example probe distribution. Here, the measured action times during training have a mean of 5.563 s (mean AT) and a standard deviation of 1.513 s (std AT). Probes are sampled from a normal distribution with a mean of mean AT - std AT - 0.5 and a standard deviation equal to std AT. The sampled probe onsets follow a truncated normal distribution within the interval of 0.5s before the average action time plus and minus one standard deviation.

The auditory probe consisted of a short “beep” that was created in Matlab ^3^. The probe had a frequency of 1200 Hz and duration of 0.04 s. Matsuhashi and Hallett (2008) state that “Tones were applied pseudo-randomly at intervals of 3–20 s, controlled by one of the investigators in a way that was not predictable by the subjects” (pp. 2345–2346). However, because further details on the exact timing of the probes are missing, these probes times are not replicable. For this reason we designed our own probe distribution. The timing of our probes are pre-determined on the basis of 25 *control* trials that were collected during a training block at the start of the experiment. The probe onsets ranged from 0.5 s before the average action time plus and minus one standard deviation. Within this interval, auditory probes followed a truncated normal distribution such that most probes were presented before the average action time (see Figure 4). A minimum probe interval of 3 s before the average action time was ensured. Moreover, the probe interval was ensured to start at least 3 s after trial onset. As well as being explicable, this probe distribution was designed to optimize experimental efficiency by ensuring that approximately one third of all trials would present a probe within 3 s before movement onset (during which awareness of an intention to act is most likely to occur). Both the *sound* and *probe* conditions used the exact same probe distribution per participant. Every trial in the *sound* or *probe* conditions could contain maximally one probe. Depending on the participants action time, this probe may or may not be presented on a certain trial.

The color feedback was slightly random. The fixation cross turned red (i.e., too fast) if the button press was made within the first 5 s after trial start + a random time interval between 0 and 3s. The fixation cross turned blue (i.e., too slow) if the button press occurs more than 10 s after trial start + a random time interval between 0 to 3 s. In all other cases where a button press was made, the fixation cross turned green. This means that the boundaries between each feedback color were blurred by 0–3 s. These blurred boundaries within the color feedback were designed to encourage the element of spontaneity. They made it difficult for a participant to count time or otherwise plan their actions to perform them at a “correct” time. Instead, they needed to refrain from planning and focus on their intention to act within the learned window of opportunity.

The presentation of the fixation cross marked the start of a trial. Each trial had a maximum duration of 9 to 14 s. The exact trial duration was chosen randomly at the start of each trial. A trial ended either because the participant had pressed the button or because the maximum trial duration was reached. The inter trial interval was chosen randomly between 1.5 and 3 s on each trial.

### 3.4. Experimental Timeline

At the start of the experiment, participants performed two training blocks. The first block consisted of 20 *control* trials and was used to train participants on the main task of pressing a button whenever they wanted to. This block was repeated until the participant performed the desired actions at roughly the right time interval. The second block consisted of 25 *control* trials and was used to collect the action times required to set the time distribution of the probes. The remainder of the experiment consisted of 4 test sequences. Each test sequence consisted of 4 blocks of 25 trials of each condition in a random order. The type of condition was displayed to the participant prior to each sequence of 25 trials of a single condition. In total, 100 trials were acquired per condition.

The experiment took 1.5 to 2 hours + 0.5 hours for setting up the EEG, which resulted in a maximum total duration of 2.5hours.

### 3.5. Behavioral Data

Three behavioral measurements were collected during the experiment: (1) the introspection reports, (2) the timing of the performed actions and (3) the answers to the questionnaire. In the *introspect* condition, the introspection reports consisted of the experienced vividness of the intention to act. In the *probe* condition they consisted of a behavioral response to a probe (a veto or ignore) and its confirmation at the end of a trial. The action timing was measured using the button presses and the onset of muscle activation as recorded with an EMG.

In order to quantify hypotheses 3, 4 and 6 (see Section 2), we needed to determine whether the probes in the *probe* condition lead to veto responses across a consistent time range prior to action onset. In other words: is there a gap in the distribution of ignored probes, as illustrated in Figure 2A and found by Matsuhashi and Hallett (2008)? To answer this question, the timing of the ignored and scheduled probes relative to action onset was analyzed. The distribution of scheduled probes refers to the average amount of probes that should by design occur prior to action onset, as described in Section 3.3. Since participants performed self-paced actions during the experiment, one could not predict their exact action onsets. Therefore, the amount of probes that were actually presented prior to action onset will always differ a bit from the scheduled ones. The distribution of ignored probes is a sample from the scheduled probe distribution that shows how many of the scheduled probes were actually presented and ignored by the participant.

To determine the distribution of scheduled probes relative to action onset, the scheduled probe onsets that precede each individual action were sampled per participant. Subsequently, the action onset was subtracted from each corresponding sampled probe onset in order to calculate the probe timing relative to action onset. A histogram with 33 time bins of 150 ms, running from 5 s prior to action until action onset, was constructed of the scheduled probe timings. In order to get an estimate of the average number of scheduled probes per participant, the histogram of scheduled probes was divided by the total number of performed actions. In addition, a histogram was created of the ignored probe times (probes that were followed by an action at a later point in time) using identical time bins. Lastly, the mean distribution of scheduled and ignored probes was calculated across all participants. A Wilcoxon signed-rank test (Wilcoxon, 1945) was used to assess whether the values of each time bin differ significantly between the ignored and scheduled probes across participants. The alpha-level for significance was Bonferroni corrected and set at 0.05/33 = 0.0015. The consecutive time points at which the number of ignored probes were found to be significantly less than the number of scheduled probes define a time range during which participants were on average aware of their intention to act (see **Figure 6**). As a control, these steps were repeated for the *sound* condition (which should not show a gap in the distribution of ignored probes prior to action onset).

The EMG measurement served to check the accuracy of the button presses. The EMG was recorded using two electrodes, placed in a bipolar pair on the right wrist and the center of the right forearm (on the flexor pollicis longus). For analysis, the EMG data of the bipolar electrodes was subtracted and band-pass filtered between 51 and 250 Hz. Subsequently, the absolute value was taken and the data was sliced in epochs of 4 s prior to a button press until 1 s after. For each participant, the average EMG activity was calculated across all trials. From this average the median and standard deviation were calculated for each participant over all time points. An individual threshold for muscle activation was set at the median EMG activity plus 2x its standard deviation. The average onset of muscle activity was determined as the point in time at which the average EMG activity exceeded the set threshold.

The mean and standard deviation of the action times relative to trial start were calculated for each participant. To quantify hypotheses 1, 2, and 5 (see Section 2), a two-factor within-subject repeated measures ANOVA was used to assess any significant effects of the manipulated variables on the mean and standard deviation of the action times between conditions. The two factors are: (1) the requirement of an introspection report and (2) the presence of probes. By using a Bonferroni correction for these two factors and their potential interaction, the significance level was set to 0.025/3 = 0.008 for a two-sided significance test. When a main effect of either manipulated variable was found, individual *post-hoc* paired-samples t-tests were used to assess specific differences in action mean or standard deviation between conditions. The significance level of these individual tests was set to 0.025 for a two-sided significance test.

In addition, the relation between probes and actions was investigated by looking at differences between conditions in action times relative to probe onset. In order to calculate the action times of the *control* and *introspect* conditions relative to probe onset, the same probe distribution was used as presented in the *sound* condition. These simulated probe onsets—button press times represent the absence of a connection between probes and actions in the *control* and *introspect* conditions and served as a control for the *sound* condition in which such a relation may be present. Differences in action mean or standard deviation relative to probe onset were assessed using a paired-sample *t*-test on all three possible combinations of the *control, introspect* and *sound* conditions. The significance level was set to 0.025/3 = 0.008. The *probe* condition was left out of this analysis since it would differ by design from all other conditions due to the performed veto responses: creating a potential gap in the distribution of ignored probes, as illustrated in Figure 2A.

### 3.6. Brain Data

EEG data was collected using 64 Ag/AgCl active electrodes sampled at 512 Hz using Biosemi equipment ^4^. The electrodes were placed according to the International 10/20 system. The electrodes measured all frequencies between 0 and 512 Hz. Two electrodes were placed on the left and right mastoids and four electro-oculogram (EOG) electrodes were placed in bipolar pairs above and below the left eye and on the outer sides of both eyes. The neural data was analyzed using Fieldtrip ^5^.

The EEG data was preprocessed using the following steps:

1. Data was sliced in epochs of 10 s before to 5 s after action onset (i.e., button press), so the data was time locked to action onset (at 0 s).
2. Data is downsampled to 256 Hz.
3. Trials in which the participant acted within 4 s after trial start were removed to ensure a decent baseline period.
4. Trials in which a probe was presented between 4.5 and 2.5 s before action onset were removed to ensure a decent baseline period.
5. Data of all conditions was concatenated per participant.
6. Data was rereferenced using a linked-mastoid reference.
7. Baseline correction was performed per trial and electrode by subtracting the average EEG signal between 3.5 and 2.5 s prior to action onset.
8. EOG artifacts were removed using a linear decorrelation of the recorded EEG and EOG (Gratton, 1998).
9. A band-pass filter between 0.2 and 47 Hz was used to filter out slow drifts and 50 Hz line noise.
10. Epochs of -5 to 3 s around action onset were retained.
11. Bad channels were removed if they differed more than 3.5 standard deviation in power from the median across all channels.
12. Bad epochs were removed if they differed more than 3.5 standard deviation in power from the median across all trials.
13. Bad channel rejection was repeated.
14. A spherical spline interpolation was used to reconstruct bad channels (Perrin et al., 1989).

To quantify hypotheses 1 and 2 (see Section 2), individual Event-Related Potentials (ERPs) were calculated per participant and per condition. To assess the main effects of the requirement of an introspection report and the presence of probes on the RP, mean ERPs were calculated across the following conditions:

1. *Control* and *sound*: providing information about all conditions without introspection reports.
2. *Introspect* and *probe*: providing information about all conditions with introspection reports.
3. *Control* and *introspect*: providing information about all conditions without probes.
4. *Sound* and *probe*: providing information about all conditions with probes.

Two within-subject cluster permutation tests with 1000 permutations were used to assess whether the last 2.5 s of data prior to action onset differed between these grouped conditions: introspection vs. no introspection and no probe vs. probe conditions (Maris and Oostenveld, 2007). After a Bonferroni correction, the significance level was set to 0.025/2 = 0.013 for a two-sided significance test. If significant main effects were found, *post-hoc* within-subject cluster permutation tests were used to identify significant differences between individual experimental conditions. The significance level of these individual tests was set to .025 for a two-sided significance test.

**Figure 5:**
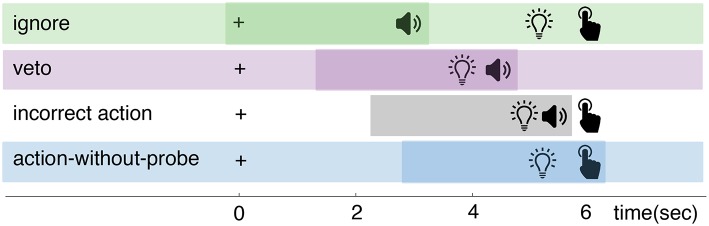
Schematic timeline of events that could happen within a trial of the *probe* condition. The “+” indicates trial start. Each *probe* trial could develop in one of four ways. (1) Ignore: a probe is presented when the participant is not intending to act. The participant ignores the probe and presses the button at a later point in time when he/she feels the intention to do so. (2) Veto: a probe is presented when the participant experiences an intention to act. The participant vetos his/her action and waits for the trial to end. (3) Incorrect action: a probe is presented when the participant experiences an intention to act. The probe happens so close to action onset, that the participant is unable to veto his/her action and presses the button anyway. (4) Action-without-probe: the participant presses a button when he/she feels the intention to do so. They are uninterrupted by a probe. The darker colored portions of each row indicate the portion of the trial that is used for EEG analysis. This portion is either time-locked to probe (ignore, veto and incorrect action trials) or action onset (action-without-probe trials).

Data from the *probe* condition was used to analyze the RP prior to ignored and vetoed probes, since this was the only condition that contained both ignore and veto responses to probes. From this data, four types of trials were extracted (see Figure 5):

1. *Action-without-probe*: trials in which no probe occurred and an action was performed at least 4 s after trial start (to ensure a decent baseline).
2. *Ignore*: trials in which a probe occurred at least 4 s after trial start and was followed by an action more than 0.5 s later (which was determined as the point of no return, as shown in Section 4.1.2).
3. *Veto*: trials in which a probe occurred at least 4 s after trial start and was followed by a veto (i.e., no action). The veto response was confirmed by the answer to the veto question at the end of the trial.
4. *Incorrect action*: trials in which a probe occurred at least 4 s after trial start and was followed by an action within 0.5 s.

**Figure 6:**
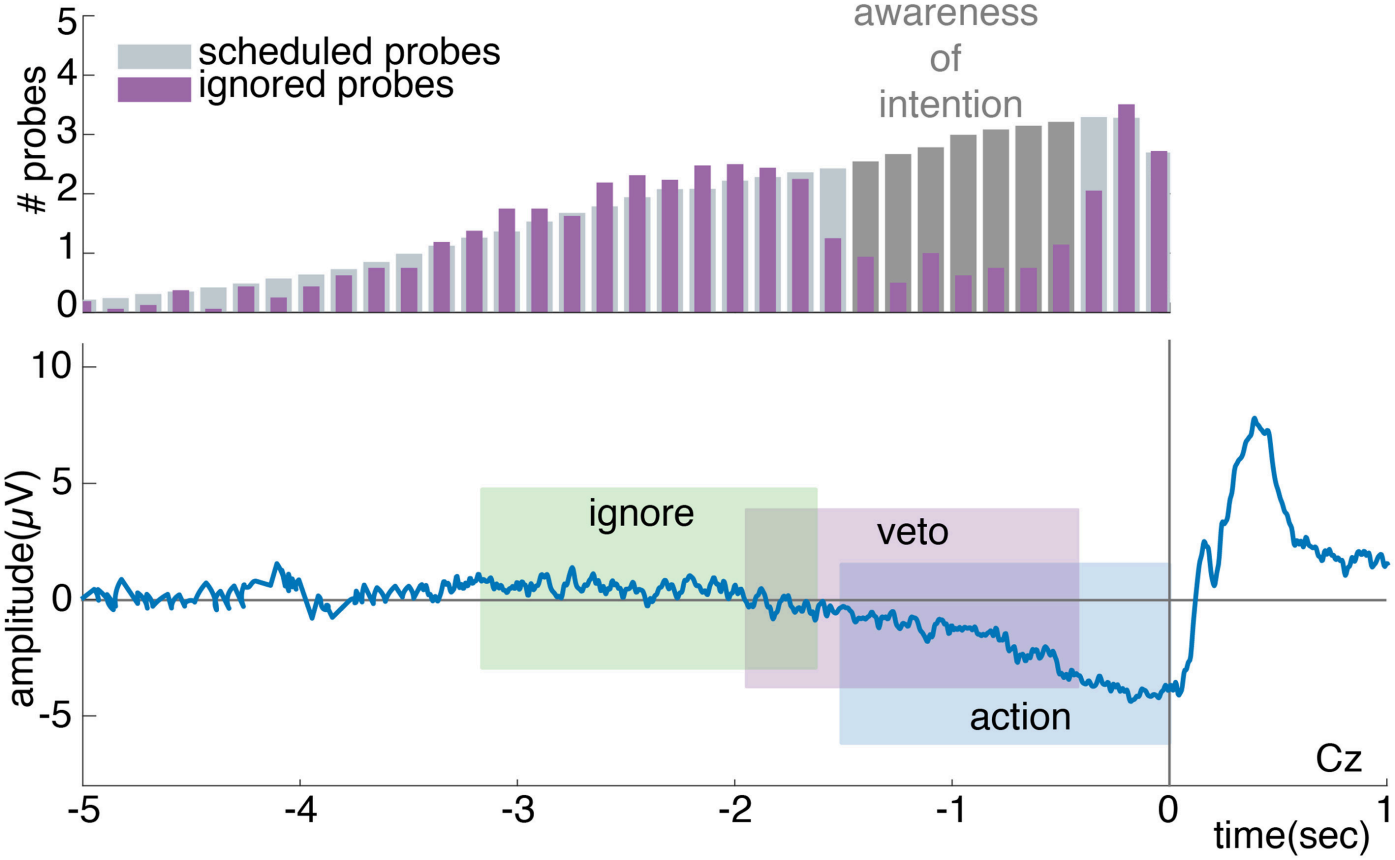
The top plot shows a schematic grand average of the distribution of scheduled and ignored probes relative to action onset. The difference between the scheduled and ignored probe distribution, as highlighted in dark gray, shows the time points at which participants are expected to perform a veto in response to a probe. The gap provides an estimated time period during which participants are expected to be aware of their intention to act. The bottom plot shows a schematic grand average of the expected RP. When looking at the average brain activity prior to an action, a full RP is expected to be present. When looking at the average brain activity prior to an ignored probe, no RP is expected. The analysis window prior to a vetoed probe is expected to sample the early phase of the RP, which given the shape of the RP will look like a weaker version of the full RP.

According to Matsuhashi and Hallett (2008), vetos should consistently be performed across a specific time range prior to action onset. Moreover, this time range should coincide with the build-up of the RP (Verbaarschot et al., 2016). If this is the case, we expect to find a weak RP signature prior to vetoed probes and no RP prior to ignored probes (see Figure 6). To test this, action-without-probe trials were time-locked to the performed button press, whereas the ignore, veto and incorrect action trials were time-locked to probe onset. To extract the general signature of the RP (i.e., a negative potential relative to baseline), the mean activities during the last 1.5 s to 0.5 s and 0.5 s to 0 s prior to action or probe onset were calculated per participant at electrode Cz. These two time intervals were used to assess whether there are differences in the early and/or late phase of the RP between these different trial types (Shibasaki and Hallett, 2006). For the two time intervals, two-sided dependent samples t-tests were used to test whether the activity differs significantly between action-without-probe and incorrect action trials and action-without-probe and veto trials (Bonferroni corrected at 0.025/8 = 0.003). A further one-sided dependent samples *t*-tests were used to test for both time intervals whether the activity was more negative in veto and action-without-probe trials relative to ignore trials (Bonferroni corrected at 0.05/8 = 0.006).

To quantify hypotheses 1 and 2 (see Section 2), individual alpha/beta ERDs were assessed. For this reason, the data of all conditions was concatenated and a spectogram was calculated between –5 and 3 s around action onset. Frequencies of interest were defined from 5 to 30 Hz using 2 Hz bins. A flexible Hanning window was used such that it included at least 7 cycles of each frequency of interest. The data was baselined using a relative baseline, resulting in the relative signal change compared to baseline (where a value of 1 means no change). The baseline activity was defined per electrode, frequency and trial as the median power between 3.5 and 2.5 s prior to action. Subsequently, the data was separated into the different conditions. The ERD was calculated per participant by taking the median power across trials for each electrode, frequency and trial. Mean ERDs were calculated per participant for no introspection (*control* and *sound*) vs. introspection conditions (*introspect* and *probe*), and no probe (*control* and *introspect*) vs. probe conditions (i.e., *sound* and *probe*). Within-subject cluster permutation tests (Maris and Oostenveld, 2007) with 1000 permutations were used to assess whether the last 2.5 s of data prior to action onset differed between the no introspection vs. introspection and no probe vs. probe conditions. After a Bonferroni correction, the significance level was set to 0.025/2 = 0.013. When a significant main effect was found, *post-hoc* within-subject cluster permutation tests were used to identify significant differences between individual experimental conditions. The significance level of these individual tests was set to .025 for a two-sided significance test.

## 4. Results

The raw data supporting the conclusions of this manuscript will be made available by the authors, without undue reservation, to any qualified researcher.

### 4.1. Behavioral Data

#### 4.1.1. Questionnaire

A total of 14 participants completed the questionnaire at the end of the experiment (see Section 3.2). Due to the exclusion of some participants (see Section 3.1), the answers of a total of 12 participants were analyzed. All 12 participants indicated that the differences between the *control, sound, introspect* and *probe* conditions were clear. Eleven participants indicated that they acted spontaneously during the entire experiment, i.e., they pressed the button as soon as they wanted to. In contrast, 1 participant indicated that he/she did not act spontaneously, i.e., he/she already decided to act long before they pressed the button.

Six participants always experienced a clear difference in timing between an intention and action, whereas 1 participant only experienced this sometimes and 1 other participant only had this experience when he/she perceived the intention consciously and vividly. One participant experienced the intention as always occurring prior to the action. Three participants did not experience any difference in timing between the intention and action and perceived them as occurring at the same time.

Concerning the *sound* and *probe* conditions, 7 participants reported the probes as neutral: they did not experience any positive or negative effects caused by the probes. However, 5 participants experienced some negative effects from the probes as they reported them to be “annoying” (2 participants), “stressful” (2 participants), or as “disturbing their relaxed state” (1 participant). Moreover, 9 participants believe that the probes did influence their course of action, whereas 3 participants did not experience any influence of the probes.

During the *probe* condition, 8 participants found it *sometimes* difficult to judge whether or not they were experiencing an intention to act when a probe was presented. One participant always experienced this intention assessment as difficult and 3 participants had no trouble with it at all. Eight participants followed instructions correctly and vetoed their intended movement when they experienced an intention to act upon probe presentation, whereas another 2 participants did not use any particular strategy to decide whether or not they could press the button after a probe was presented. Two participants determined whether or not they should veto their act based on their expectation of a probe. Whenever they were expecting a probe and a probe was presented, they would veto their subsequent act. In their case, the performed veto's did not relate to their intention to act but to their expectation of a probe. Because these participants were effectively not following instructions correctly, they were excluded from further analysis (as indicated in Section 2.1).

In summary, 92% of the participants who completed the questionnaire acted spontaneously throughout the experiment. Although 75% of the participants at least sometimes perceived their intentions and actions as two different events in time, 75% of participants also found it difficult to assess upon probe presentation whether or not they were intending to act. Lastly, the probes seem to be experienced in a negative way by at least 42% of participants.

#### 4.1.2. Intention Reports

**Figure 7:**
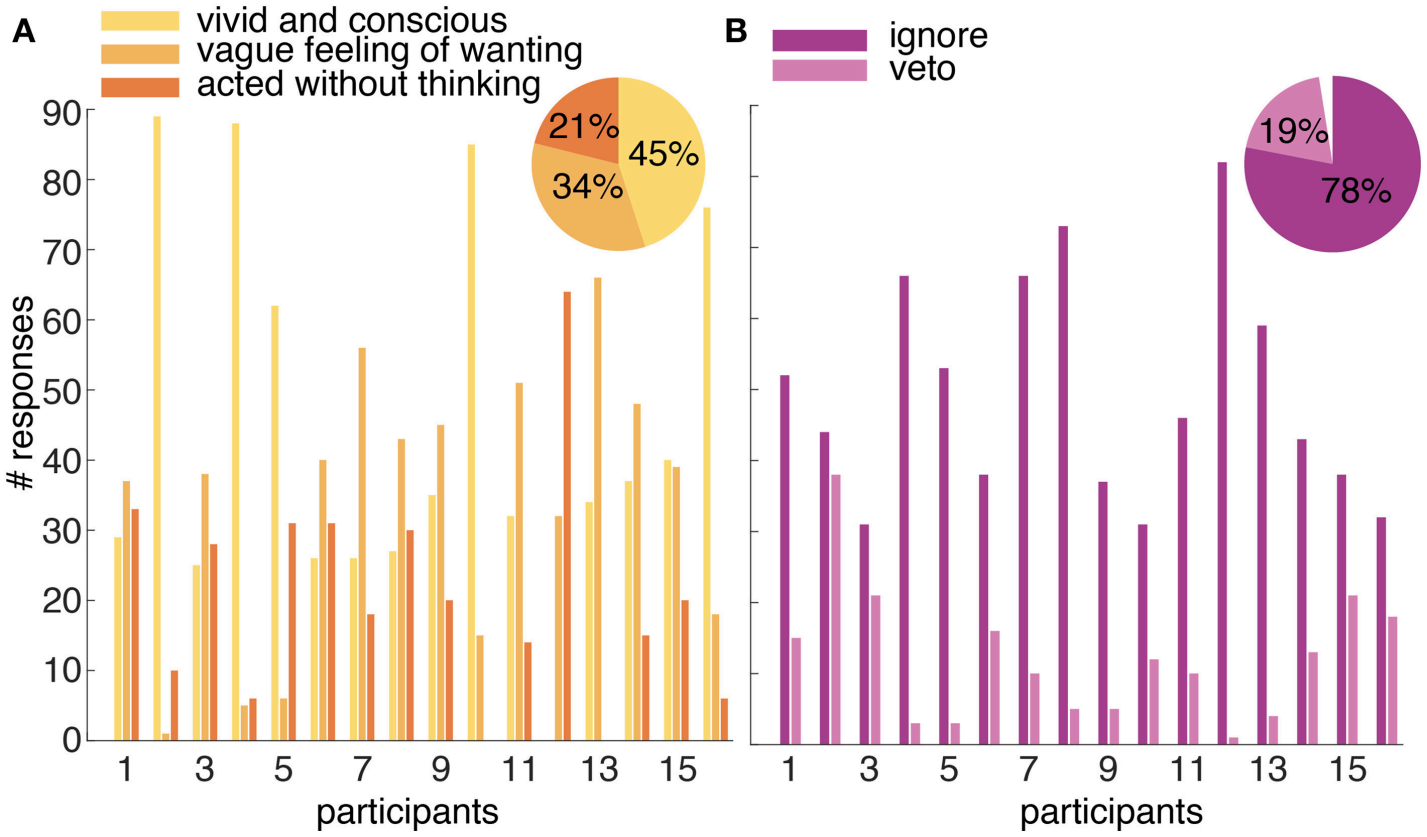
**(A)** Reported experience of an intention to act in the *introspect* condition. Participants could report their intention to act after every trial as “vivid and conscious,” “a vague feeling of wanting,” or “pressed the button without thinking about it.” The number of times each possible answer was selected is shown per participant. The percentage of each selected answer across all participants is shown in the pie chart in the top right corner. **(B)** Overview of the responses to a probe in the *probe* condition. The number of times a presented probe is followed by an ignore or a veto response is shown for each participant. The percentage of presented probes that were followed by an ignore or veto response across all participants is shown in the pie chart in the top right corner. The gap in the pie chart shows the percentage of presented probes that were ignored but not followed by an action (i.e., the participant did not intend to act during the trial).

Each participant completed 100 trials of the *introspect* condition. Twenty-three (=1%) trials across all participants ended without a button press. Participants reported having experienced their intention as “vivid and conscious” in 45% (±2%) of all trials containing an action. In 34% (±1%) the intention was experienced as a “vague feeling of wanting.” In the remaining 21% (±1%), participants reported to have “pressed the button without thinking about it.” Figure 7A shows the reports on the subjective experience of intending to act for each participant and across all participants.

Similar to the *introspect* condition, 100 trials were collected per participant in the *probe* condition. In 63% (±1%) of all trials across participants, a probe was presented. Seventy-eight% (±16%) of the presented probes across participants were followed by an ignore response, whereas 20% (±14%) was followed by a veto response. Three % (±5%) of the presented probes were followed neither by an ignore or a veto response; the trial simply ended before an action was made. Figure 7B shows the number of ignore and veto responses for each participant and the percentage of these responses across all participants.

**Figure 8:**
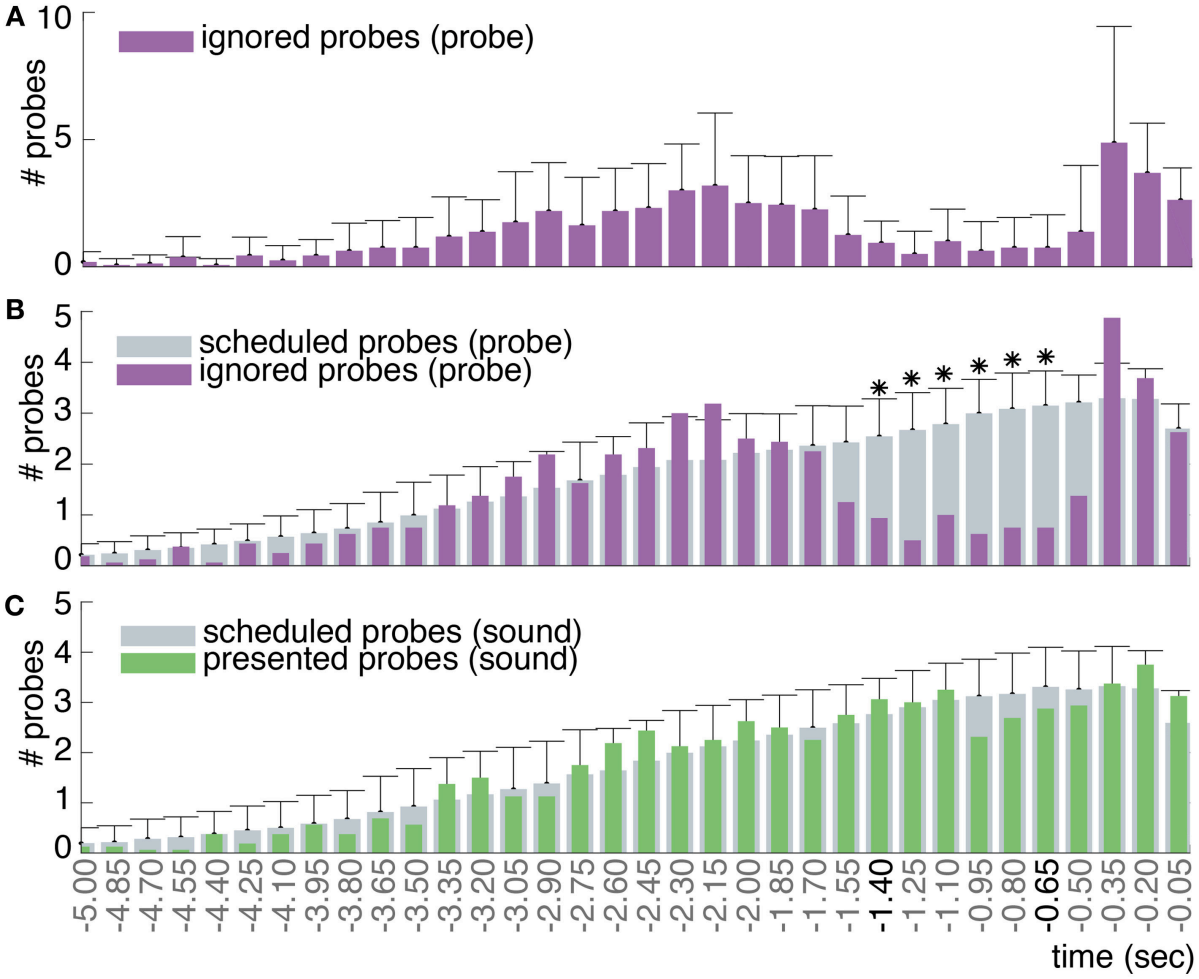
**(A)** The mean and standard deviation of the ignored probes across all participants in the *probe* condition. **(B)** In gray the mean number of probes that were scheduled to be presented prior to action across all participants in the *probe* condition (standard deviations across participants are provided on top of the histogram). In purple the mean number of probes that were ignored and followed by an action (at time 0) across all participants. Around –1.4 s prior to action, participants start to veto their action in response to a probe: this is apparent by the decrease in the observed fraction of probes that were followed by an action compared to the scheduled fraction of probes. Around –0.5 s prior to action, participants are no longer able to veto their action in response to a probe due to their close proximity in time (i.e., point of no return), hence the increase in ignored probes. Note: the purple distribution of ignored probes in **(B)** is identical to that in **(A)**, but with a different scaling factor. Significant (*p* < 0.0015) differences between the amount of scheduled and ignored probes are indicated with an asterisk (*). **(C)** In gray the mean number of probes that were scheduled to be presented prior to action across all participants in the *sound* condition. In green the mean number of probes that were actually presented and followed by an action (at time 0) across all participants.

Figures 8A,B show the number of observed ignored probes and scheduled probes relative to action onset across all participants for the *probe* and *sound* conditions. As noted in Section 3.5, the distribution of observed ignored probes will always differ a bit from the distribution of scheduled probes since the scheduled probes are an approximation of the observed probes: i.e., the predicted amount of probes that on average should occur prior to action onset. Furthermore, the distribution of ignored probes includes the amount of probes that are presented and followed by an action only, whereas the distribution of scheduled probes also includes probes that are not followed by an action (i.e., a veto).

Since our probes do not follow a uniform but a truncated normal distribution, our plot looks slightly different from those of Matsuhashi and Hallett (2008). Similar to Matsuhashi and Hallett (2008), we observe a significant decrease in the fraction of ignored probes between 1.4 and 0.65 s prior to action (*p* < 0.0015) for the *probe* condition. During this time period, participants mostly performed a veto in response to a presented probe. Shortly prior to action, from 0.5 s to action onset, the fraction of ignored probes increases again. This increase could be due to the point of no return as defined by Matsuhashi and Hallett (2008). At the point of no return, a probe was presented in such close proximity to action onset that participants are not able to cancel their action anymore to perform a veto. As shown in Figure 8C, the distribution of presented probes of the *sound* condition also shows a slight decrease in the amount of probes between 1 and 0.5s prior to action. However, this deviation between the scheduled and presented probes was not found to be significant.

#### 4.1.3. Action Distribution

Across all participants, the mean onset of muscle activation was found at 81 ms (±78*ms*) prior to a button press. Since this difference is small relative to the magnitude of the expected intention reports (about 1 s) and estimation errors, the timing of the button press is used as action onset throughout the analysis.

**Figure 9:**
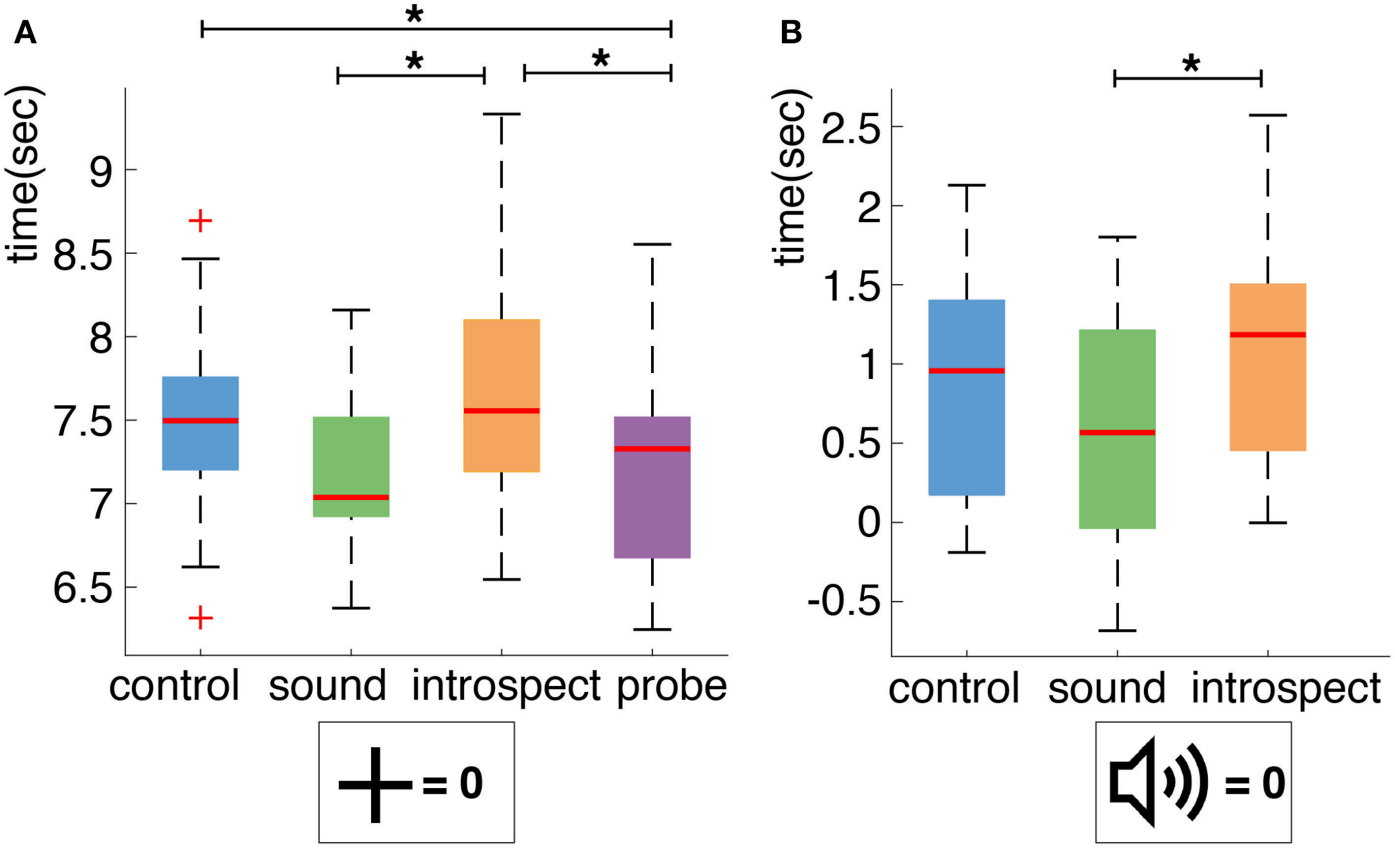
**(A)** Boxplot of action times per condition across all participants. Action times are measured relative to trial start. **(B)** Boxplot of action times per condition across all participants. Action times are measured relative to probe presentation. Significant differences (*p* < 0.025 for **A** and *p* < 0.008 for **B**) between conditions are indicated with an asterisk (*).

A boxplot of the action times of the *control* (mean: 7.501 s, standard deviation: 1.549 s), *sound* (mean: 7.180 s, standard deviation: 1.447 s), *introspect* (mean: 7.720 s, standard deviation: 1.618 s) and *probe* (mean: 7.179 s, standard deviation: 1.493 s) conditions across all participants is shown in Figure 9A. A significant main effect of the presence of probes on the mean action time was found (*df* = 15, *F* = 15.704, *p* = 0.0013). Individual *post-hoc* tests reveal that this is due to significant differences between the *probe* and *control* (*df* = 15, *t* = −3.100, *p* = 0.007), *sound* and *introspect* (*df* = 15, *t* = 3.823, *p* = 0.002), and *probe* and *introspect* conditions (*df* = 15, *t* = −4.199, *p* =.000). No significant main effect of the requirement of an introspection report on mean action time was found (*df* = 15, *F* = 3.291, *p* = 0.090). Furthermore, no significant main effects of the requirement of an introspection report or the presence of probes was found on the standard deviation of action times.

Furthermore, the effect of the presence of a probe on action timing was explicitly investigated by comparing the action timings relative to probe onset across all conditions. A boxplot of the action timings relative to probe onset of the *control* (mean: 0.886 s, standard deviation: 1.801 s), *sound* (mean: 0.574 s, standard deviation: 1.704 s) and *introspect* (mean: 1.110 s, standard deviation: 1.840 s) conditions across all participants is shown in Figure 9B. Again, the mean action time of the *sound* condition was found to differ significantly from that of the *introspect* (*df* = 15, *t* = 3.8226, *p* =.002^*^) condition. No significant differences in mean action time were found between the *control* and *introspect* or *control* and *sound* conditions. No significant differences in the standard deviation of action times were found between any of the conditions.

### 4.2. Brain Data

After preprocessing, 85 (minimum: 40, maximum: 91) trials remained for analysis of the *control* condition, 85 (minimum: 46, maximum: 92) trials for the *sound*, 87 (minimum: 48, maximum: 96) trials for the *introspect* and 75 (minimum: 28, maximum: 89) trials for the *probe* condition.

#### 4.2.1. Readiness Potential

**Figure 10:**
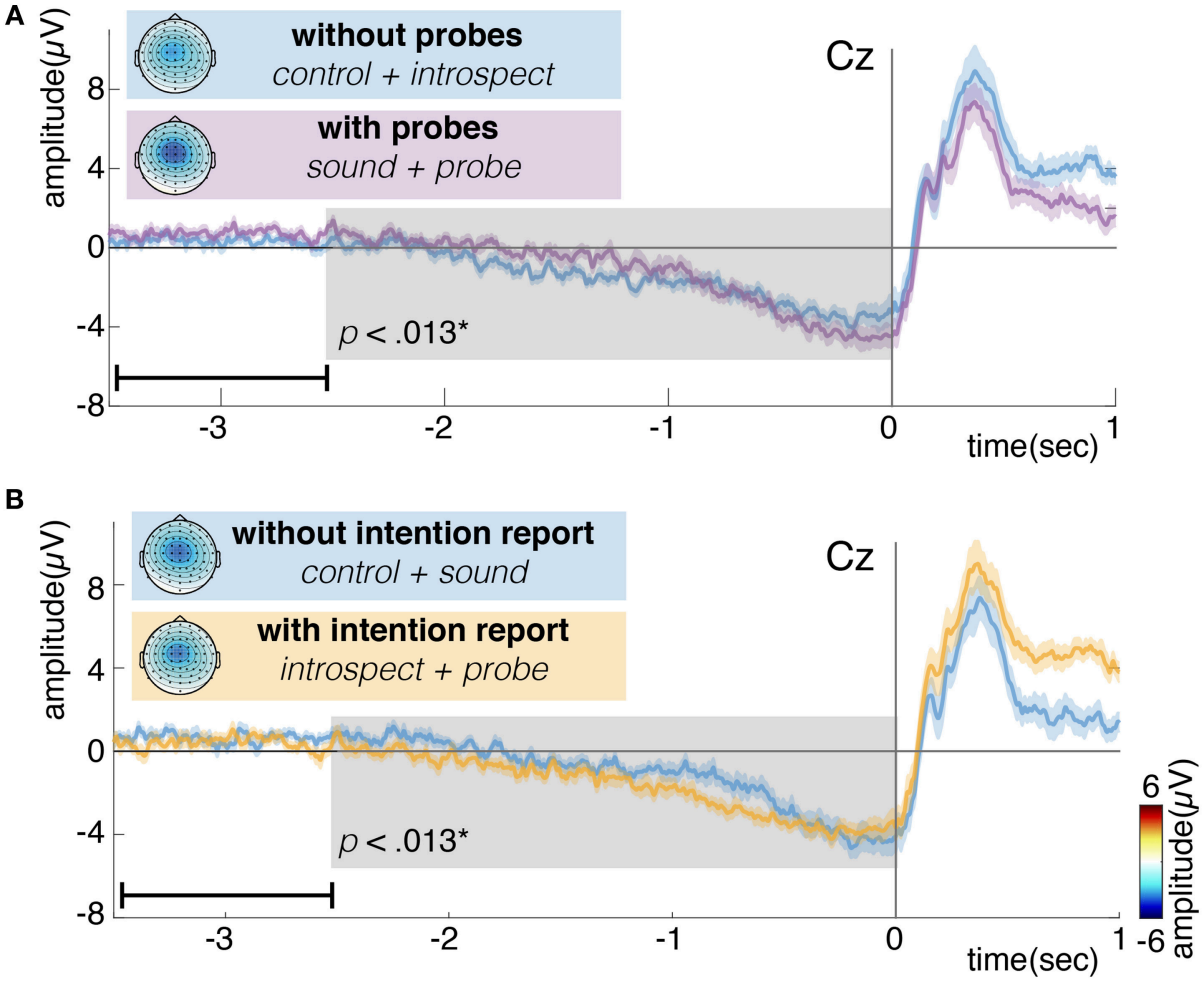
**(A)** Grand average readiness potential at electrode Cz for conditions without (control + introspect) and with probes (sound + probe). **(B)** Grand average readiness potential at electrode Cz for conditions without (control + sound) and with intention reports (introspect + probe). Action onset is at time 0 and is indicated by a vertical line. The colored shade indicates the standard error across participants. The topoplots show the grand average EEG activity at each electrode, averaged across the last 0.5s prior to action onset. Significant differences (*p* < 0.013) are indicated by a gray box. Note, the data is baselined between 3.5 and 2.5 s prior to action, as indicated by a small horizontal line in the bottom left corner of each plot.

**Figure 11:**
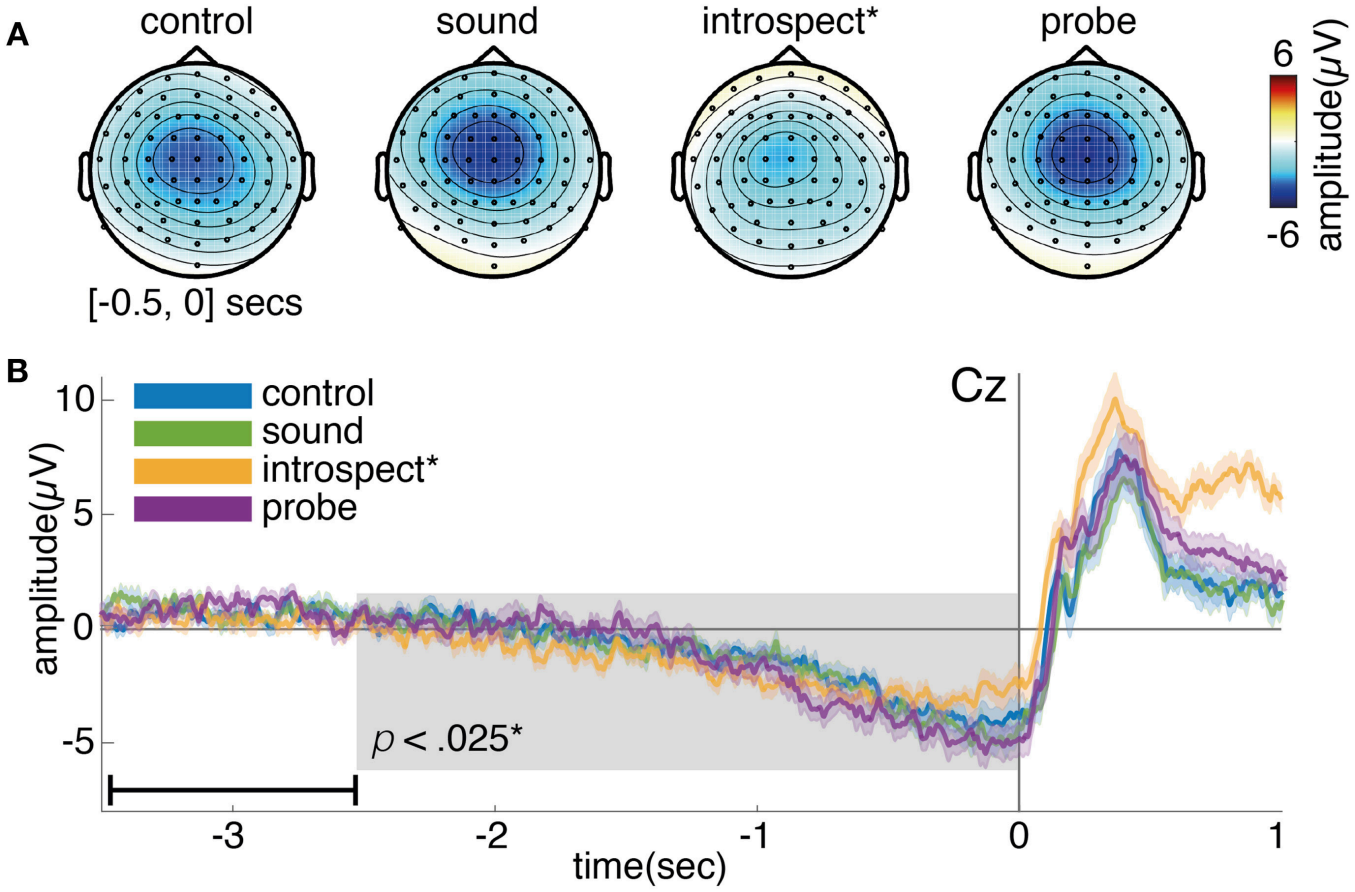
**(A)** Topoplot showing the grand average EEG activity at each electrode, averaged across the last 0.5s prior to action onset. **(B)** Grand average of the readiness potential at electrode Cz. Each color shows the grand average RP of a single condition surrounded by two lines, indicating the standard error across participants. Action onset is at time 0 and is indicated by a vertical line. Note, the data is baselined between 3.5 and 2.5 s prior to action, as indicated by a small horizontal line in the bottom left corner of plot B. The introspect condition was found to differ significantly from all others (*p* < 0.025).

Figure 10 shows the grand average of the RP for conditions with and without introspection reports and with and without probes. Visually, the RP seems to have its earliest onset around 2 s prior to movement. The shape and timing of the RP confirm previous research involving spontaneous voluntary right hand movements (Kornhuber and Deecke, 1965; Libet et al., 1983; Shibasaki and Hallett, 2006). Significant main effects of the requirement of an introspection report and the presence of probes are found on the last 2.5 s of the RP (*N* = 16, *p* < 0.008, see Figure 10). Whereas probes seem to cause a slight increase in RP amplitude, the requirement of an introspection report seems to cause a slight decrease in RP amplitude. The *introspect* condition seems to lie at the heart of these effects, as the RPs in this condition were found to differ significantly from those in all other conditions (*N* = 16, *p* < 0.025). Figure 11 shows the grand average of the RP for each individual condition.

After action onset, the RP of the *introspect* condition deviates from the others (see Figure 11). This is due to a difference in events after action onset: immediately after action performance in the *introspect* condition, participants are prompted with a question about the vividness of their intention to act and need to respond to this question by pressing one of three buttons.

**Figure 12:**
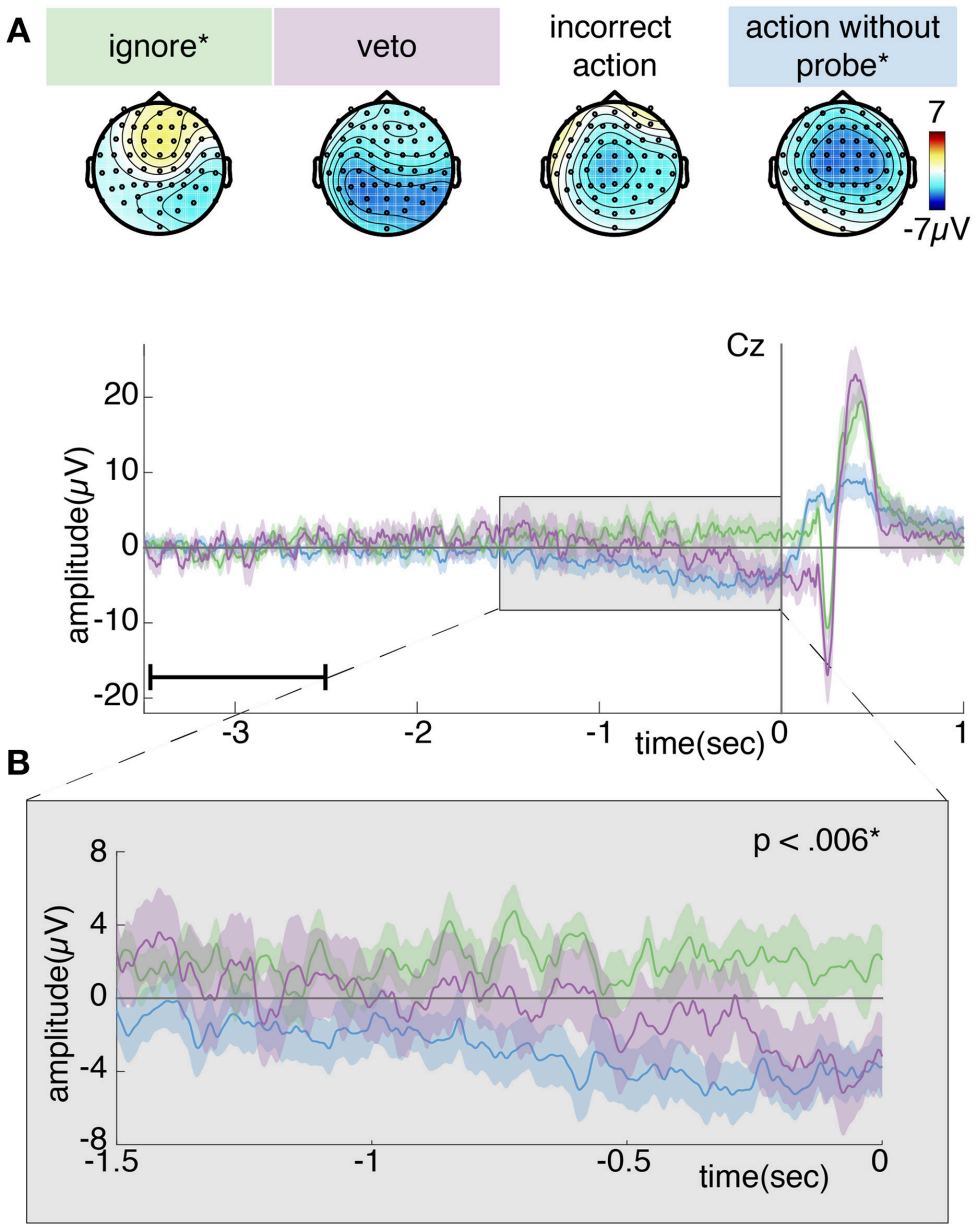
**(A)** Topoplot showing the grand average EEG activity of the probe condition averaged across the last 0.5s prior to action (action-without-probe trials) or probe onset (ignore, veto, or incorrect action trials). **(B)** Grand average ERP at electrode Cz. Each color shows the grand average RP of a single trial type surrounded by the standard error across participants. For simplicity, we leave out the incorrect trials, since they closely resemble the action-without-probe trials. Time 0 corresponds to action (action-without-probe trials) or probe (ignore or veto) onset and is indicated by a vertical line. A significant difference between action-without-probe and ignore trials was found across –1.5 to 0 s. Note, the data is baselined between 3.5 and 2.5 s prior to action or probe onset, as indicated by a small horizontal line in the bottom left corner of plot **(B)**.

Concerning the *post-hoc* analysis of the RP prior to vetoed and ignored probes, Figure 12 shows the grand average ERP for different trials of the probe condition. The number of ignored and vetoed probes differ greatly among participants within this condition (see Section 4.1.2). Especially the number of vetoed probes is quite low: around 20%. For this reason, we removed some participants from further analysis: only those participants who retained at least 10 trials action-without-probe, ignore and veto trials were kept for further analysis. This resulted in a total of 8 participants containing on average 39 (minimum: 30, maximum: 53) action-without-probe trials, 25 (minimum: 19, maximum: 32) ignore trials, 15 (minimum: 10, maximum: 20) veto trials and 11 (minimum: 5, maximum: 18) incorrect action trials per participant.

Figure 12A shows a clear negativity across the motor cortex for the action-without-probe and incorrect action trials. This is to be expected since both trial types include an intended action and thus should look similar to the RP in Figure 11. The incorrect action trials are a weaker version of the action-without-probe trials because they contain less data and are time-locked to probe onset rather than action onset. As the RP increases in amplitude up to action at 0 s, time-locking to earlier times in the RP development results in reduced ERP amplitudes, as seen here for the incorrect action trials where probes occur randomly somewhere in the last 0.5 s before action onset. The veto trials show a medium and more widespread negativity across the motor cortex, whereas this negativity seems completely absent for ignore trials. A similar trend can be observed from Figure 12B: a clear negativity (i.e., RP) can be observed for action-without-probe trials, whereas ignore trials remain around baseline. Veto trials (which we expect are randomly sampled from –1.4 to –0.65 s before action based on the intention-window identified in Figure 8) are in between ignore and action-without-probe trials, showing a medium negativity shortly prior to probe onset.

One-sided dependent samples *t*-tests showed that the action-without-probe trials are significantly higher in amplitude compared to the ignore trials during both the early (–1.5 to –0.5 s: *df* = 7, *t* = −3.5217, *p* =.005) and late (–0.5 to 0 s: *df* = 7, *t* = −5.4562, *p* = 0.001) phase of the RP. No significant differences were found between the action-without-probe and incorrect action trials (*df* = 7, *t* = −0.3285, *p* = 0.752), action-without-probe and veto trials (*df* = 7, *t* = −1.928, *p* = 0.095) or veto and ignore trials (*df* = 7, *t* = −1.014, *p* = 0.172) for the early phase of the RP. Similarly, no significant differences were found between the action-without-probe and incorrect action trials (*df* = 7, *t* = −0.4593, *p* = 0.670), action-without-probe and veto trials (*df* = 7, *t* = −1.969, *p* = 0.090) or veto and ignore trials (*df* = 7, *t* = −2.587, *p* = 0.018) for the late phase of the RP.

Figure 12 also shows differences between action-without-probe, ignore, veto and incorrect action trials after time 0s. Note however, that time 0 s refers to action onset for action-without-probe trials only and to probe onset for ignore, veto and incorrect action trials. Therefore, any differences after time 0s likely reflect the presence of an action or probe (or the combination of the two for incorrect action trials). We do not investigate brain activity after time 0 s because any differences in brain processing related to action preparation will be contaminated by the brain response to probe presentation or action performance.

#### 4.2.2. Alpha/Beta Event-Related Desynchronization

**Figure 13:**
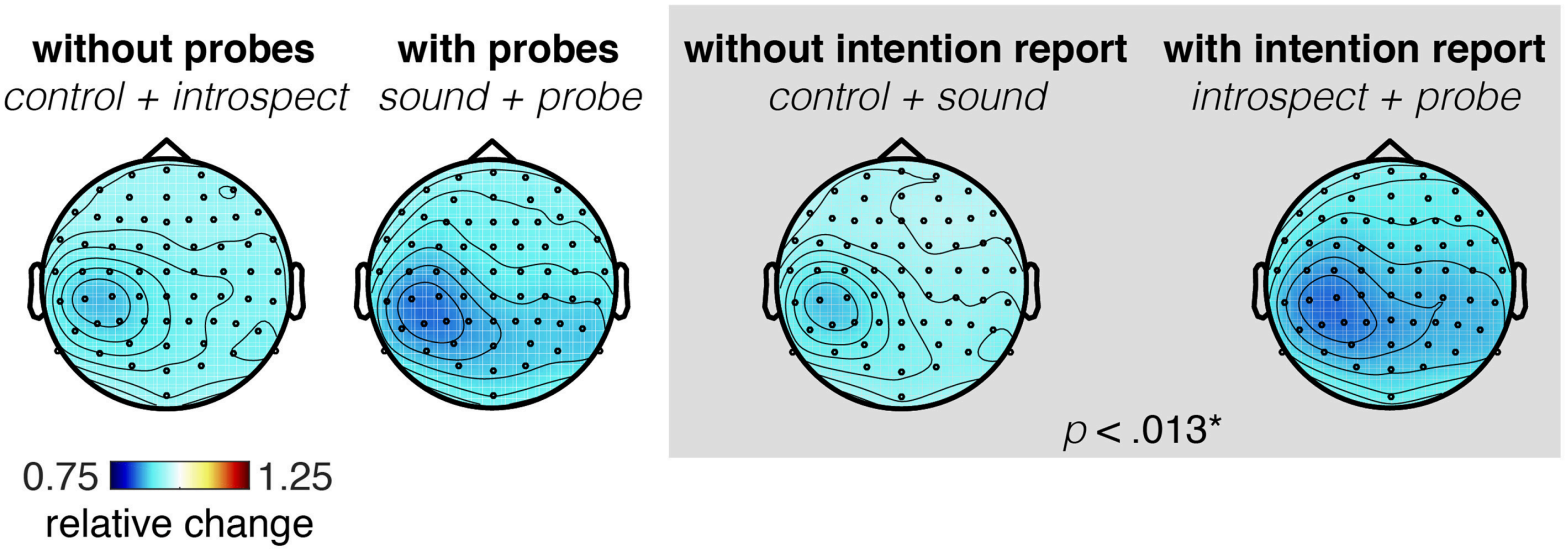
Topoplots showing the grand average power across 8–30 Hz at each electrode, averaged across the last 0.5 s prior to action onset. Averages across conditions with (sound + probe) and without probes (control + introspect), and with (introspect + probe) and without intention reports (control + sound) are shown. A significant difference (*p* < 0.013) is found between conditions with and without intention reports.

**Figure 14:**
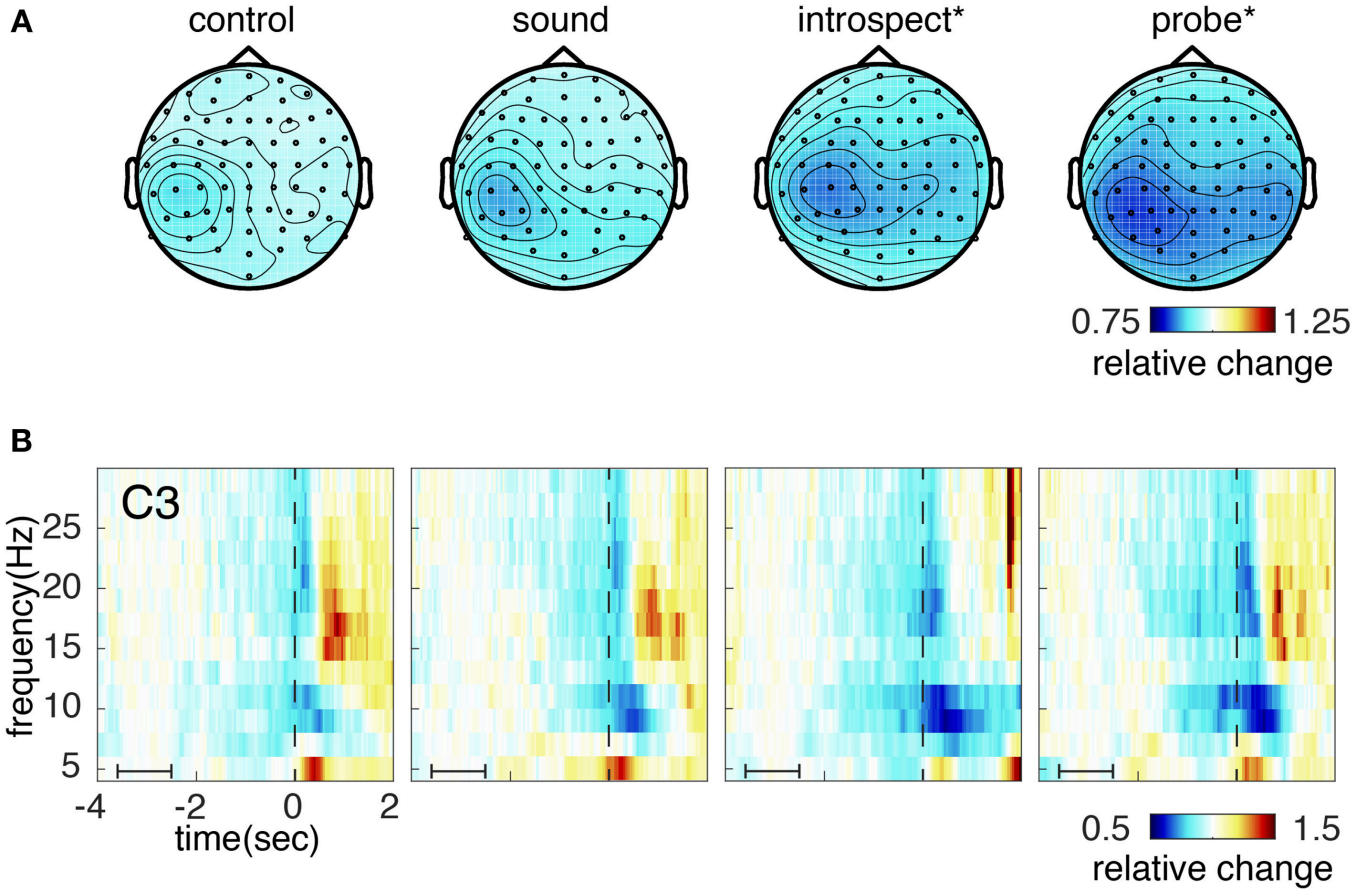
**(A)** Topoplot showing the grand average power across 8-30Hz at each electrode, averaged across the last 0.5s prior to action onset. **(B)** Grand average of the event-related desynchronization across the alpha (8–1 2Hz) and beta (13–30 Hz) bands measured at electrode C3. Each spectogram corresponds to the grand average data of an individual condition: *control* (left), *sound* (left of middle), *introspect* (right of middle) and *probe* (right). The dotted vertical line indicates action onset (time 0). The data is baselined between 3.5 and 2.5 s prior to action, as indicated by the horizontal line in the bottom left corner.

Significant main effects of the requirement of an introspection report and the presence of probes are found on the last 2.5 s of the RP (*N* = 16, *p* < 0.013, see Figure 13). Figure 14 shows the grand average of the ERD across the alpha (8–12 Hz) and beta (13–30 Hz) bands at channel C3 for each condition. An ERD is visible in the average time-frequency spectrum prior and during action (time 0). The ERD seems to have its earliest onset at around 2 s prior to action onset. Judging from the topoplot provided in Figure 14A, the ERD seems enhanced in the introspect and especially probe conditions compared to the control and sound conditions. After action, an event-related synchonization is visible in the control, sound and probe conditions, whereas the ERD seems to continue after movement in the introspect condition. This continued ERD in the introspect condition is caused by the second button press that is required to answer the multiple-choice question on the subjective experience of the intention to act. Overall, the shape and timing of the observed ERD and ERS signatures confirm those of previous research (Pfurtscheller and Aranibar, 1979; Doyle et al., 2005). Individual *post-hoc* tests reveal significant differences in the ERD between the probe and sound, probe and control, and introspect and control conditions (*N* = 16, *p* < 0.025).

## 5. Discussion

The probe method of Matsuhashi and Hallett (2008) promises to be a valuable addition to the clock method of Libet et al. (1983) in studying the timing of an intention to act. Here, it was put to test to verify its accuracy and quantify any potential concerns that might limit its usage. In a 2 × 2 within-subject design (1) the requirement of an introspection report and (2) the presence of an auditory probe were manipulated. In total, the experiment consisted of 4 conditions: *control, sound, introspect* and *probe*. In all conditions, participants were instructed to make a spontaneous self-paced right hand movement between 5 and 10 s after trial start. The *control* condition consisted solely of the performance of these voluntary actions. In addition, the *sound* and *probe* conditions contained (identical) auditory probes. Furthermore, the *introspect* and *probe* conditions required a report on the experienced intention to act. In the *introspect* condition, this report was provided by answering a multiple-choice question on the vividness of an experienced intention. In the *probe* condition, it was provided through the response to a probe: ignoring the probe or vetoing the intended action. In addition, 14 participants completed a questionnaire to get more insight into their subjective experience during the experiment. The effect of the two manipulated variables on the neural preparatory activity for action (RP and alpha/beta ERD), the awareness of an intention, and the performed actions was investigated.

The presence of probes had a significant effect on the observed mean action times. This effect was due to significant differences in mean action time between the *sound* and *introspect, control* and *probe*, and *probe* and *introspect* conditions. Regardless of the requirement of an introspection report, the presence of probes seem to speed up the mean action time by roughly 0.3 s. One possible explanation for this observation is that the action times get tuned toward the probe distribution. The probe distribution is designed to be a shifted version of the action distribution. On average, this shift causes the probes to be presented slightly prior to the actions. This precedence of probes could bias the actions by speeding them up. Although a significant difference is found between the *control* and *probe* conditions, it was not found between the *control* and *sound* condition. On the one hand, this may be due to the fact that the sampling of actions in the *probe* condition slightly enhances the effect of the probes: slow actions are more likely to be turned into a veto response due to a probe. Because these slow actions will not be performed, they do not add to the mean action time. This enhances the difference between mean action onset in the *probe* compared to the *sound* condition. On the other hand, this result may also confirm hypothesis 5: veto influences action. Participants may act faster in the *probe* condition because they want to either explicitly (consciously) or implicitly (subconsciously) decrease the chance of having to perform a veto.

When specifically investigating the effect of the probes on the action times, the mean action times relative to probe onset of the *sound* condition were found to differ from those of the *introspect* condition. However, the action times relative to probe onset did not differ significantly between the *sound* and *control* nor the *introspect* and *control* conditions, suggesting that the difference between the *sound* and *introspect* conditions are a combined effect from the relatively slower action times in the *introspect* condition and faster action times of the *sound* condition.

Although probes seem to speed up actions, they do not seem to speed up brain signals. In contrast, it is not the presence of probes, but the requirement of an introspection report that seems to influence brain signals: reducing the amplitude of the RP and increasing the desynchronization in the alpha and beta bands prior to action onset. Specifically, the RPs in the *introspect* condition have the lowest amplitude of all. This may suggest that the introspection requirement influences the threshold for action performance, making one more susceptible to small fluctuations in neural activity. Moreover, performing introspection may enhance the level of attention, which reduces the general level of alpha activity and could cause a relatively enhanced desynchronization in this range. These types of effects of introspection, i.e., tuning attention toward ones internal state, have been reported to induce changes in brain activity in the past (Lau et al., 2007).

Nine out of 12 participants who completed the questionnaire indicated that they found it difficult to assess whether or not they were intending to act upon probe presentation in the *probe* condition. Yet, vetos were performed in a specific time range across all participants: running from 1.4 until 0.65 s prior to action onset. Finding a significant descrease in the amount of ignored probes during this time range confirms the main behavioral findings of Matsuhashi and Hallett (2008). Moreover, within the probe condition, we found a significant difference in brain activity prior to an ignored probe and an action: we see a clear RP prior to action onset, but nothing like an RP prior to an ignored probe. This suggests that there is no neural preparatory activity for action present prior to an ignored probe, providing further evidence indicating that participants are indeed not experiencing an intention to act when they ignore a probe.

The average onset of the awareness of an intention to act found here and in previous research (Matsuhashi and Hallett, 2008; Verbaarschot et al., 2016) is much earlier than that found by studies using the clock method (Libet et al., 1983; Haggard and Eimer, 1999; Haggard et al., 2002; Sirigu et al., 2004; Banks and Isham, 2009; Bode et al., 2011; Fried et al., 2011; Soon et al., 2013; Jo et al., 2014; Douglas et al., 2015; Tabu et al., 2015; Alexander et al., 2016). This suggests that probes spread out the awareness of an intention to act. During a specific time range prior to action (starting around 1.4 s before), probes seem to facilitate the awareness of an intention. Within this time range, probes are consistently followed by a veto response across all participants. As argued previously (Uithol et al., 2014; Verbaarschot et al., 2016), these findings suggests that an intention to act reflects a dynamic process rather than a discrete mental state. The probe method is able to measure the awareness of intending during the earlier stages of this process, during which one seems susceptible to external stimuli. In contrast, the clock method seems to measure only its final stage, during which intentions become available for self-initiated report.

The probe distribution is designed to present the majority of probes in the last 3s prior to action onset, where awareness of an intention is most likely to occur (Matsuhashi and Hallett, 2008; Verbaarschot et al., 2016). The choice of probe distribution may co-determine the results: awareness of an intention to act starting earlier than 3s prior to action onset may be missed using our probe distribution. We believe our choice of probe distribution should be sufficient since our results as well as those of Matsuhashi and Hallett and Verbaarschot et al. show that probes are consistently followed by a veto response starting from 1.5s prior to action onset, and not earlier. If needed, the probe distribution could be extended in future research to investigate earlier awareness of an intention to act. If one would want to measure the timing of an intention to act across a larger time period, one would need to extend the experiment in order to retain a similar time resolution: a fixed amount of probes with small variations in timing provides a more detailed approximation of the timing of an intention to act during a limited time period, than the same amount of probes with big variations in timing across a larger time period.

Eleven out of 12 participants who completed the questionnaire indicated that they acted spontaneously throughout the experiment. Although participants were instructed to act whenever they experienced an intention to do so, only 45% of these intentions were reported as vivid and conscious across all participants in the *introspect* condition. The remaining intentions were either perceived as a vague feeling of wanting (34%) or without any conscious thought at all (21%). These results might be related to the type of task that participants were asked to perform: an arbitrary button press that is performed without any reason or consequence. In daily life, these actions are usually not preceded by a vivid intention. This type of task has been criticized in the past (Mele, 2010; Nachev and Hacker, 2014) and highlights the importance of designing an ecologically valid experiment that aims to measure meaningful actions (Mecacci and Haselager, 2015). In contrast to the clock method, the probe method can be used to reach this goal as it does not require constant introspection and can be used in combination with other stimuli. Additional stimuli can be used to create an ecologically valid experimental context in which actions can be made for a reason and have some consequence.

We used trial-by-trial color feedback rather than a sporadic verbal correction [as most probably used by Matsuhashi and Hallett (2008)] to prevent potential instructional differences between participants. In addition, the feedback was designed to provide an intuitive feeling of the time window of opportunity for acting without the need to keep track of time. However, the feedback may have enhanced the artificial nature of the experimental task and decreased the element of spontaneity in comparison to that of Matsuhashi and Hallett (2008). In a previous study we conducted a Matsuhashi style experiment without the use of color feedback (Verbaarschot et al., 2016). Participants had complete freedom to decide when to act and what action (a left or right hand button press) to perform. In our current study, we observed a similar action pattern to that of our previous Matsuhashi style experiment in terms of action mean and variance across all conditions. As we believe that action variance increases with spontaneity, this suggests that the actions measured in this experiment are at least as spontaneous as those measured in our previous Matsuhashi type experiment that did not use any color feedback. Moreover, since participants acted around 5-7 s after trial start in the previous Matsuhashi experiment, the 5–10 s window of opportunity used in the current experiment does not seem to restrict the average participant's spontaneous actions in an obvious way (Verleger et al., 2016). In future research, we encourage the use of a more ecologically valid task that evokes the required action timing and pattern in a natural and intuitive way without the use of explicit feedback.

In summary, our test of the probe method has confirmed certain effects of the presence of probes and the requirement of an introspection report: probes speed up actions and introspection changes the neural preparation for action. However, we do not believe that these effects make the probe method an unsuitable alternative to the clock method to study the timing of intentions to act. The RP and alpha/beta ERD were clearly detectable in all experimental conditions, confirming previous findings on the neural preparation for a voluntary movement (Pfurtscheller and Aranibar, 1979; Libet et al., 1983; Shibasaki and Hallett, 2006). Moreover, vetos were performed consistently across participants from 1.4 to 0.65 s prior to action onset. Together with previous research, this time range has been confirmed in three independent investigations (Matsuhashi and Hallett, 2008; Verbaarschot et al., 2016). Probes do seem to affect the timing of actions, speeding them up a bit (about 300 ms on average compared to the *control* condition). But they do not seem to speed up or delay brain signals. The requirement of an introspection report does influence the brain signals. However, this effect is common to both the probe and clock methods, as they both require introspection reports. Moreover, the continuous introspection, as required by the clock method and mimicked by the *introspect* condition, seems to exacerbate these effects as shown by the lower amplitude RP and enhanced desynchronization in the alpha and beta bands prior to action onset.

We believe that the probe method provides a valuable addition to the clock method. The probe method can be used in combination with other tactile, visual, or even auditory (if the probe is easily detectable) stimuli, creating the possibility to embed it in a more realistic and ecologically valid experimental task. By including it in our repertoire, intentional actions can be studied in various experimental contexts. In contrast to the clock method, the probe method measures the awareness of an intention to act in real-time during action preparation. As such, it requires only sporadic introspection. Moreover, the probe method seems capable of measuring earlier stages of intending compared to the clock method. Depending on one's research objective, one might favor the clock or probe method over the other. When the amount of experimental time needs to be limited and one is interested in the onset of a reportable intention to act, one might best opt for the clock method. On the other hand, when devising a complex and ecologically valid experimental task and one is interested in the time period during which one is aware of an intention to act, the probe method seems the best way to go. With this overview and our current findings, we hope to encourage the use of Matsuhashi and Hallet's probe method in future research and extend the repertoire for experimentally studying intended action.

## Author Contributions

CV was involved in all stages of the research. In addition, she performed the data collection and analysis. All authors contributed equally to the experimental design. PH was mainly involved in the formation of research questions, the conceptual analysis and interpretation of the data. JF contributed mainly to the methods, data analysis and interpretation of subsequent results.

### Conflict of Interest Statement

The authors declare that the research was conducted in the absence of any commercial or financial relationships that could be construed as a potential conflict of interest.

## Footnotes

^1^We note that similar criticisms could be raised for the moving clock stimulus used in Libet-type studies.

^2^see www.brainstream.nu

^3^see www.mathworks.com

^4^see www.biosemi.com

^5^see www.fieldtriptoolbox.org
